# Bacterial Hypoxic Responses Revealed as Critical Determinants of the Host-Pathogen Outcome by TnSeq Analysis of *Staphylococcus aureus* Invasive Infection

**DOI:** 10.1371/journal.ppat.1005341

**Published:** 2015-12-18

**Authors:** Aimee D. Wilde, Daniel J. Snyder, Nicole E. Putnam, Michael D. Valentino, Neal D. Hammer, Zachery R. Lonergan, Scott A. Hinger, Esar E. Aysanoa, Catlyn Blanchard, Paul M. Dunman, Gregory A. Wasserman, John Chen, Bo Shopsin, Michael S. Gilmore, Eric P. Skaar, James E. Cassat

**Affiliations:** 1 Department of Pathology, Microbiology, and Immunology, Vanderbilt University Medical Center, Nashville, Tennessee, United States of America; 2 Departments of Ophthalmology and Microbiology and Immunology, Harvard Medical School, Boston, Massachusetts, United States of America; 3 Department of Biological Sciences, Vanderbilt University, Nashville, Tennessee, United States of America; 4 Department of Microbiology and Immunology, University of Rochester Medical Center, Rochester, New York, United States of America; 5 Departments of Medicine and Microbiology, New York University School of Medicine, New York, New York, United States of America; 6 Skirball Institute Program in Molecular Pathogenesis, Departments of Microbiology and Medicine, New York University Medical Center, New York, New York, United States of America; 7 Veterans Affairs Tennessee Valley Healthcare Services, Nashville, Tennessee, United States of America; 8 Department of Pediatrics, Division of Pediatric Infectious Diseases, Vanderbilt University Medical Center, Nashville, Tennessee, United States of America; National Institutes of Health, UNITED STATES

## Abstract

*Staphylococcus aureus* is capable of infecting nearly every organ in the human body. In order to infiltrate and thrive in such diverse host tissues, staphylococci must possess remarkable flexibility in both metabolic and virulence programs. To investigate the genetic requirements for bacterial survival during invasive infection, we performed a transposon sequencing (TnSeq) analysis of *S*. *aureus* during experimental osteomyelitis. TnSeq identified 65 genes essential for staphylococcal survival in infected bone and an additional 148 mutants with compromised fitness *in vivo*. Among the loci essential for *in vivo* survival was SrrAB, a staphylococcal two-component system previously reported to coordinate hypoxic and nitrosative stress responses *in vitro*. Healthy bone is intrinsically hypoxic, and intravital oxygen monitoring revealed further decreases in skeletal oxygen concentrations upon *S*. *aureus* infection. The fitness of an *srrAB* mutant during osteomyelitis was significantly increased by depletion of neutrophils, suggesting that neutrophils impose hypoxic and/or nitrosative stresses on invading bacteria. To more globally evaluate staphylococcal responses to changing oxygenation, we examined quorum sensing and virulence factor production in staphylococci grown under aerobic or hypoxic conditions. Hypoxic growth resulted in a profound increase in quorum sensing-dependent toxin production, and a concomitant increase in cytotoxicity toward mammalian cells. Moreover, aerobic growth limited quorum sensing and cytotoxicity in an SrrAB-dependent manner, suggesting a mechanism by which *S*. *aureus* modulates quorum sensing and toxin production in response to environmental oxygenation. Collectively, our results demonstrate that bacterial hypoxic responses are key determinants of the staphylococcal-host interaction.

## Author Summary

*Staphylococcus aureus* is a leading cause of infectious death, yet is also capable of harmlessly colonizing healthy individuals. These disparate observations imply that *S*. *aureus* can modulate its growth and virulence in response to different host environments. To characterize the staphylococcal genetic programs required to sustain invasive infection, we applied a technique known as TnSeq to experimental osteomyelitis. Osteomyelitis is one of the most common invasive manifestations of staphylococcal infection, and a better understanding of the bacterial factors required to colonize and destroy bone will aid in vaccine and antimicrobial development. TnSeq identified more than 200 genes important for invasive staphylococcal infection of bone. Two of these genes encode a bacterial two-component system, SrrAB, which is known to help *S*. *aureus* survive in low oxygen. Consistent with this finding, we discovered that oxygen levels in bone decrease during osteomyelitis. Furthermore, we discovered that staphylococcal virulence is augmented by environmental oxygen levels, suggesting one strategy by which *S*. *aureus* can respond to different host environments. Collectively, our results define the genetic and metabolic programs required for *S*. *aureus* to sustain invasive infection.

## Introduction

*Staphylococcus aureus* is a major human pathogen, capable of causing a variety of life-threatening, invasive diseases and infecting nearly every organ in the human body. Yet *S*. *aureus* also innocuously colonizes the skin and nares of one-third to one-half of the population [1]. These facts suggest a remarkable flexibility in terms of metabolic and virulence programs, allowing staphylococci to adapt to diverse and changing host environments during invasive infection, while also enabling a commensal lifestyle characterized by low virulence and immunotolerance. The mechanisms by which bacterial pathogens adapt to changing host environments are poorly understood, in part due to the technical difficulty in measuring adaptive responses *in vivo*. However, recent advances in high-throughput sequencing have enabled an unprecedented evaluation of the host-pathogen interface. Transposon sequencing (TnSeq) is a sensitive and high-throughput tool combining highly-saturated transposon mutant libraries with massively-parallel sequencing to calculate the fitness of all nonessential bacterial genes under a given selective pressure [2]. TnSeq has been successfully used to determine the bacterial genes required for survival in a number of different *in vitro* conditions and infection models [3–7]. More recently, a TnSeq library was generated in *S*. *aureus* and used to determine genes contributing to fitness in abscess and infection-related ecologies [8]. These studies illustrate the power of TnSeq analyses to determine the genetic requirements for bacterial adaptation to diverse host environments.

One of the most common invasive disease manifestations of staphylococcal infection is osteomyelitis, and *S*. *aureus* is by far the most common pathogen causing musculoskeletal infection. Osteomyelitis causes enormous morbidity, including functional disabilities, the requirement for invasive procedures, and the propensity to evolve into chronic infection even with appropriate management [9,10]. Two factors contribute to the therapeutic recalcitrance of osteomyelitis. First, skeletal tissues are intrinsically hypoxic, and bacterial infection further disrupts the vascular architecture of bone [11–13]. Second, the human skeleton is constantly being remodeled through the opposing actions of osteoblasts and osteoclasts. The kinetics of bone remodeling are affected dramatically by bacterial infection through osteo-immunologic crosstalk [14–16]. Thus, pathogens invading the bone must adapt to hypoxia as well as constant shifts in the available host substrates for adhesion and nutrient acquisition. While these factors would seemingly create an inhospitable environment for bacterial proliferation, bone is one of the most common locations of metastatic infection following *S*. *aureus* bacteremia [17]. One mechanism by which bacterial pathogens adapt to potentially hostile host environments is through the actions of one or more two-component systems (TCSs). Bacterial TCSs consist of a membrane bound histidine kinase sensor, which upon binding of its cognate ligand phosphorylates a response regulator. Response regulators most often function as transcriptional factors, and differentially coordinate changes in gene expression in response to a given stress. We therefore hypothesized that the ability of *S*. *aureus* to adapt to changes in available oxygen and shifts in substrate availability in inflamed skeletal tissues may rely on one or more TCSs, and that these responses would be key determinants of pathogenesis during osteomyelitis.

In this study, we employed TnSeq analysis during acute murine osteomyelitis to determine the genetic requirements for *S*. *aureus* survival during invasive infection. A large number of *S*. *aureus* genes were identified as essential for growth within bone, some of which have previously been implicated in hypoxic responses. Intravital oxygen monitoring was utilized to define changes in tissue oxygenation during osteomyelitis. Finally, we evaluated the effects of changing oxygenation on *S*. *aureus* quorum sensing and virulence factor production. Collectively, these studies determine the staphylococcal genes essential for survival during invasive infection of bone, define shifts in tissue oxygenation during invasive infection, and interrogate the mechanisms by which *S*. *aureus* can modulate its virulence in response to changes in oxygen availability.

## Results

### Identification of *S*. *aureus* genes essential for invasive infection by transposon sequencing (TnSeq) analysis of experimental osteomyelitis

In order to characterize the genes required for invasive *S*. *aureus* infection, TnSeq analysis was performed during experimental osteomyelitis using a recently described *S*. *aureus* transposon insertion library in strain HG003 [8]. To identify potential bottlenecks in bacterial survival during osteomyelitis, a timecourse infection was first performed by inoculating murine femurs with strain HG003. An inoculum of 5x10^6^ CFU was chosen based on direct comparison with strain LAC, which has served as the wildtype strain in prior osteomyelitis analyses and is representative of the most common lineage (USA300) of strains causing osteomyelitis in the United States [18]. At days 1, 3, 5, 7, and 12 post-infection, infected femurs were harvested and processed for CFU enumeration. After an initial period of replication from day 1 to day 3 post-infection, decreases in bacterial burdens were noted by days 5 and 12 (**S1 Fig**). Day 5 was therefore chosen for TnSeq analysis of acute osteomyelitis, as it likely represents the first bottleneck encountered by invading bacteria. For TnSeq analysis of osteomyelitis, mice were infected with the TnSeq library by direct inoculation into the femur. Five days post-infection, femurs from infected mice were processed for genomic DNA extraction. One limitation of TnSeq analysis during invasive infection is the requirement for an outgrowth step after the recovery of bacteria from infected tissues. Although *in vitro* outgrowth could potentially confound fitness calculations, it is necessary to decrease host DNA contamination and allow for efficient sequencing of microbial DNA, and thus has become a standard practice during TnSeq analysis of invasive infection models [5,8,19–23]. We opted for a short outgrowth in liquid media to minimize any confounding effects on fitness calculation. For an *in vitro* comparator, an equivalent volume of the osteomyelitis inoculum was grown *in vitro* for 24 hours prior to collection and genomic DNA extraction. To determine mutants with compromised *in vivo* fitness, a “dval” was calculated for each gene in each condition (inoculum, *in vitro* comparator, or osteomyelitis).

A total of 65 genes were found to be essential for survival during osteomyelitis (**S1 Table**) but not *in vitro* growth, and mutations in an additional 148 genes resulted in significant *in vivo* compromise relative to the *in vitro* comparator (**S2 Table**). Of the 213 genes identified by TnSeq, 39 essential and 73 compromised genes encode hypothetical proteins, respectively. Of the remaining 101 genes, 12 essential genes and 32 compromised genes have Kyoto Encyclopedia of Genes and Genomes (KEGG) identifiers. Thirty-two of the 44 genes with KEGG identifiers can broadly be categorized into metabolic pathways, with specific pathways represented including carbon metabolism (9 genes), amino acid biosynthesis (7 genes), and the TCA cycle (5 genes). In the TCA cycle, mutations in genes *sucB* (SAOUHSC_01416), *sucC* (SAOUHSC_01216), and *sucD* (SAOUHSC_01218), which encode enzymes responsible for the conversion of α-ketoglutarate to succinate, each resulted in compromised growth during osteomyelitis. Moreover, genes encoding enzymes in pathways that feed into the TCA cycle were also important for intraosseous growth, including pyruvate carboxylase (SAOUHSC_01064 *pyc*), pyruvate dehydrogenase (SAOUHSC_01040 *pdhA*), and a putative malic enzyme (SAOUHSC_01810). Mutations in 7 *S*. *aureus* genes encoding amino acid biosynthesis enzymes compromised bacterial growth during osteomyelitis, yet did not significantly impair growth *in vitro*. These genes encode enzymes in the biosynthetic pathways for tryptophan (SAOUHSC_01369 *trpC*, SAOUHSC_01367 *trpG*, and SAOUHSC_01377), cysteine (SAOUHSC_00488 *cysK*), lysine (SAOUHSC_01868), leucine (SAOUHSC_02288 *leuD*), and the conversion of serine to glycine (SAOUHSC_02354 *glyA*). Mutations in 6 genes encoding components of purine and pyrimidine metabolic pathways resulted in significant *in vivo* compromise during osteomyelitis. Two of these genes (SAOUHSC_02126 *purB*, SAOUHSC_02360 *tdk*) were essential for staphylococcal survival in bone. A substantial portion of the oxidative phosphorylation pathway was also found to be necessary for staphylococcal growth during osteomyelitis. Four of the 12 essential genes with KEGG identifiers and 1 of the mutants with compromised growth are involved in oxidative phosphorylation, including components of quinol oxidase complexes (SAOUHSC_01000 *qoxC*, SAOUHSC_01032 *cydB*), and 3 subunits of the F-type ATPase (SAOUHSC_02340 *atpC*, SAOUHSC_02343 *atpG*, SAOUHSC_02346 *atpH*). Collectively, the results of TnSeq analysis during experimental osteomyelitis suggest broad adaptations in metabolism and energy production are required for staphylococcal survival during invasive infection of bone.

In contrast to an abundance of genes encoding hypothetical proteins or metabolic pathways, relatively few genes encoding known or putative virulence factors were identified by TnSeq as important for staphylococcal survival in bone. Phosphatidylglycerol lysyltransferase, encoded by *mprF* (SAOUHSC_01359), catalyzes the modification of phosphatidylglycerol with L-lysine and contributes to bacterial defenses against neutrophils, cationic antimicrobial peptides, and certain antibiotics [24]. The *mprF* gene was essential for growth during osteomyelitis, suggesting that resistance to antimicrobial peptides and neutrophils are important components of staphylococcal survival in bone. A second virulence-associated gene identified by TnSeq as essential for *S*. *aureus* osteomyelitis was *isdF* (SAOUHSC_01087), which encodes a component of the iron-regulated surface determinant heme uptake system [25]. Interestingly, mutation of the ferric uptake regulator (SAOUHSC_00615 *fur*) gene also resulted in compromised intraosseous growth, illustrating a potential role for iron acquisition in the pathogenesis of staphylococcal osteomyelitis. Mutation in the genes encoding thermonuclease (SAOUHSC_00818 *nuc*), a fibrinogen-binding protein (SAOUHSC_01110), the repressor of toxins (SAOUHSC_01879 *rot*), and two serine proteases (SAOUHSC_01935 *splF*, SAOUHSC_01938 *splD*) also compromised the survival of *S*. *aureus* during osteomyelitis.

The genes identified by TnSeq as critical for staphylococcal osteomyelitis encode diverse metabolic processes, hypothetical proteins, and select virulence factors. These results suggest that complex bacterial adaptations occur in response to invasive infection of bone. One mechanism by which bacterial pathogens sense and ultimately respond to host-imposed stresses is through TCSs. We therefore hypothesized that staphylococcal TCSs might coordinate the complex adaptations observed during osteomyelitis. Strikingly, TnSeq analysis identified only one *S*. *aureus* TCS as required for intraosseous survival. The staphylococcal respiratory response (SrrAB) system is involved in coordination of the staphylococcal response to hypoxia and other stresses [26], and has been shown to directly regulate select virulence factors [27]. Both the histidine kinase (*srrB*) and the response regulator (*srrA*) components of the SrrAB locus were essential for staphylococcal survival in bone, implying that this TCS might be particularly important for coordination of the metabolic and virulence adaptations to intraosseous growth (**S1 Table**). In total, these results reveal the power of TnSeq analysis to identify *S*. *aureus* genes required for invasive infection of bone.

### SrrAB differentially regulates *S*. *aureus* genes under aerobic and hypoxic growth, and is required for survival during osteomyelitis

Among the mutants that exhibited decreased survival in the osteomyelitis model, we identified a single TCS, SrrAB, which coordinates responses to hypoxia and nitrosative stress *in vitro* [26]. Moreover, mutations in two additional genes regulated by SrrAB specifically under conditions of nitrosative stress, *cydB* and *qoxC*, also resulted in significantly decreased fitness during osteomyelitis (**S1 and S2 Tables**) [26]. Bone and bone marrow are intrinsically hypoxic, leading to the hypothesis that SrrAB contributes to osteomyelitis pathogenesis by sensing and responding to changes in environmental oxygen [11,28]. Because the SrrAB regulon was previously defined under conditions of nitrosative stress, we sought to further define the oxygen-dependent SrrAB regulon by performing global transcriptional analysis of a clinically relevant strain (LAC) in comparison to a mutant strain lacking *srrAB* expression (Δ*srrA*) in both aerobic and hypoxic growth conditions. Inactivation of *srrAB* under aerobic conditions resulted in the differential regulation of 64 genes (39 transcripts increased in abundance and 25 decreased in abundance upon inactivation of *srrAB*) (**S3 Table**). Under hypoxic growth conditions, *srrAB* inactivation led to differential regulation of 78 genes (22 transcripts increased in abundance and 56 decreased in abundance) (**S4 Table**). Of the genes differentially regulated by SrrAB under aerobic or hypoxic conditions, only 16 were previously identified as members of the SrrAB regulon under nitrosative stress, suggesting that specific stresses might invoke different SrrAB-dependent transcriptional responses [26]. Moreover, by defining the SrrAB regulon under aerobic versus hypoxic conditions, we discovered that an additional 7 genes important for survival during osteomyelitis in the TnSeq dataset are also SrrAB-regulated (**S3 and S4 Tables**). The requirement of multiple genes in the SrrAB regulon for survival during osteomyelitis suggests that the SrrAB TCS is an important orchestrator of *S*. *aureus* stress responses in inflamed skeletal tissues.

Previous reports have demonstrated a significant defect in the growth of an *srrAB* mutant under anaerobic conditions but not under hypoxic growth conditions [26,27,29]. To confirm that SrrAB was not found to be essential in the TnSeq analysis simply because of a defect in growth, the Δ*srrA* polar transposon mutant and mutations in known genes of the SrrAB regulon (*pflA*, *pflB*, *qoxA*, and *qoxC*) were analyzed in the LAC strain background. The growth rate of each mutant under aerobic or hypoxic conditions was monitored over time. Under aerobic and hypoxic growth conditions, Δ*srrA* had an enhanced lag phase compared to WT but reached equivalent optical densities to WT by 8 hours (**S2 Fig**). The Δ*qoxA* and Δ*qoxC* mutants were unable to reach maximal optical densities as previously reported due to disruption of the electron transport chain [26]. These results indicate that an *srrAB* mutant is not impaired for growth under hypoxia, further validating our TnSeq methods.

**Fig 1:**
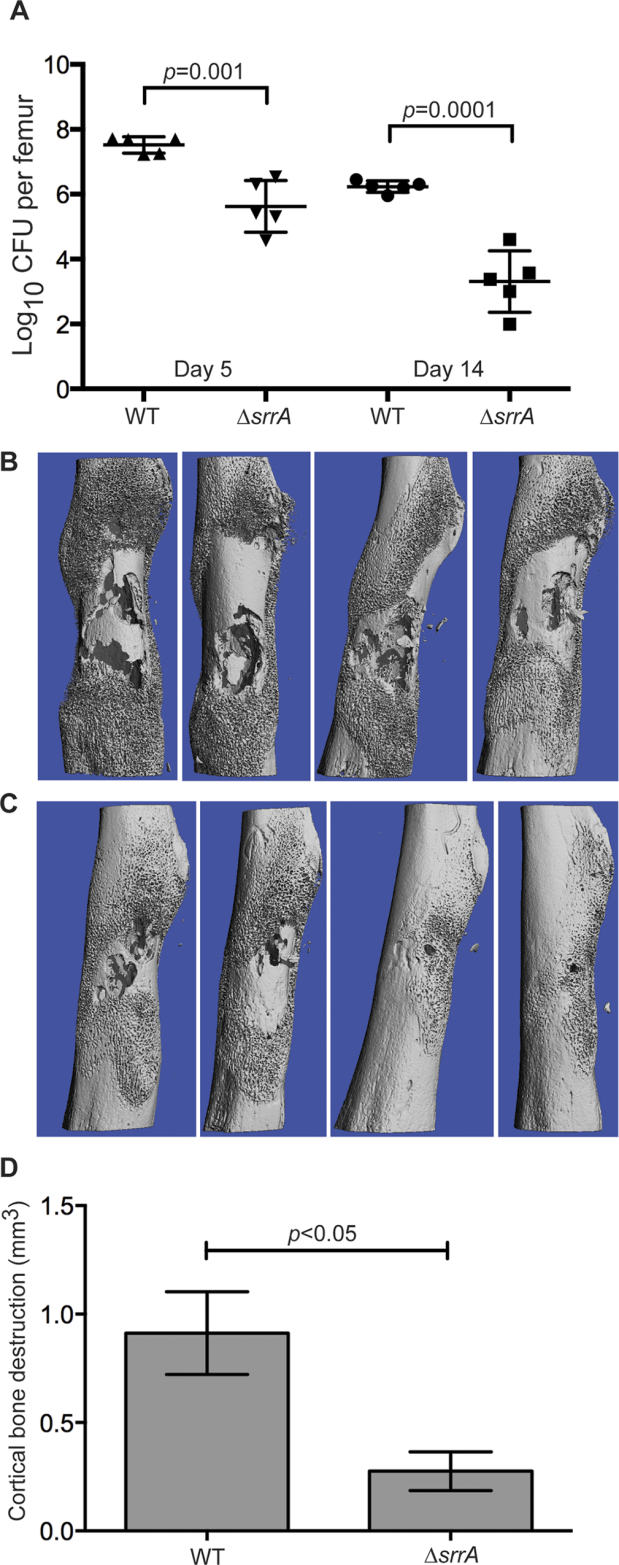
SrrAB is required for intraosseous survival and cortical bone destruction during *S*. *aureus* osteomyelitis. Osteomyelitis was induced in groups of mice using WT or Δ*srrA* strains. (A) At 5 and 14 days post-infection, femurs were processed for colony forming units (CFU) enumeration. N = 5 mice per group. Horizontal line represents the mean and error bars represent SD. (B and C) Antero-posterior views of WT (B) or Δ*srrA* (C) infected femurs at 14 days post-inoculation. (D) MicroCT imaging analysis of cortical bone destruction (mm^3^) 14 days post-inoculation. N = 4 mice per group. Error bars represent the SEM. Statistical significance determined by Students *t* test.

To investigate the role of SrrAB in osteomyelitis in a clinically-relevant background without the potentially confounding influence of competition from other mutants in the TnSeq library, groups of mice were infected with either WT or Δ*srrA* in the LAC background. At 5 or 14 days post-infection, femurs were either processed to quantify bacterial burdens or subjected to micro-computed tomography (microCT) imaging (day 14) for quantification of cortical bone destruction. Inactivation of SrrAB resulted in a significant reduction in bacterial burdens in infected femurs at both 5 and 14 days post-infection (**Fig 1A**). Moreover, murine femurs infected with Δ*srrA* sustain significantly less cortical bone destruction than WT-infected femurs (**Fig 1B–1D**). These results demonstrate that SrrAB is critical for *S*. *aureus* survival in infected bone and for induction of pathologic changes in bone remodeling during osteomyelitis. Furthermore, the data suggest that staphylococci encounter hypoxic and/or nitrosative stresses during osteomyelitis.

### *S*. *aureus* osteomyelitis triggers reduced oxygen availability in skeletal tissues

**Fig 2:**
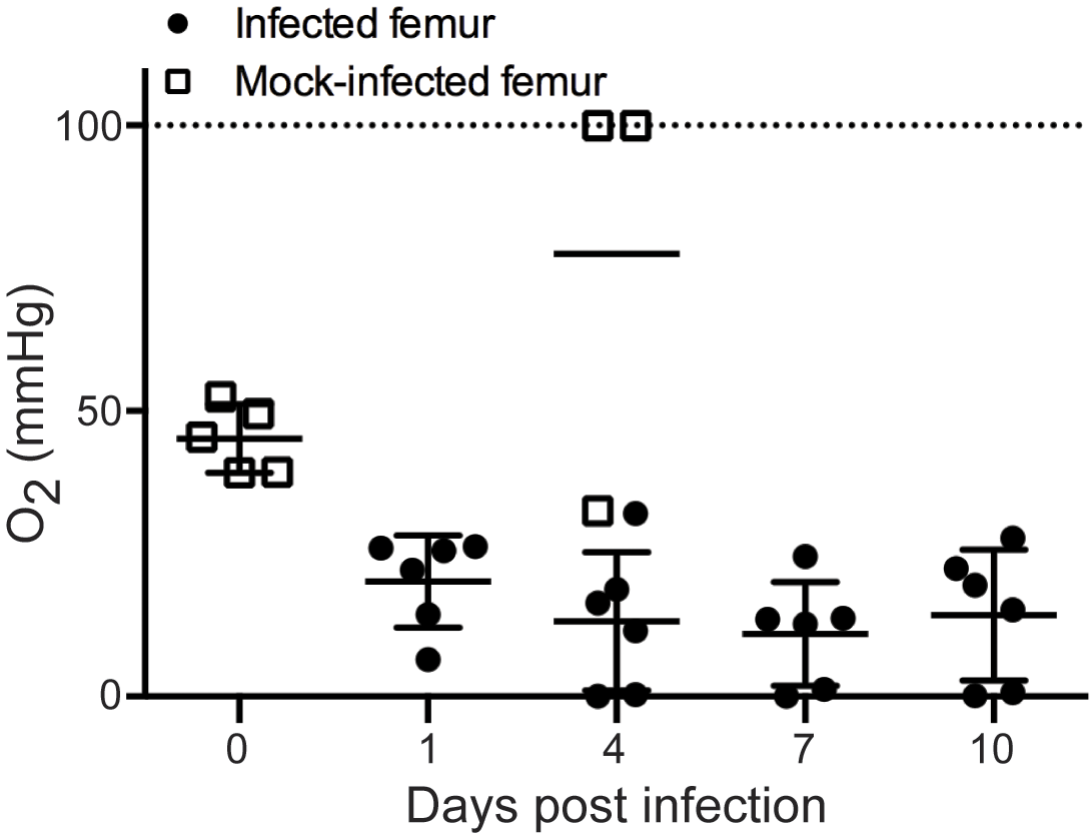
*S*. *aureus* osteomyelitis triggers reduced oxygen availability in skeletal tissues. Oxygen tension (pO_2_) was measured in murine femurs infected with *S*. *aureus* (black circles) at 1, 4, 7 and 10 days post-infection (n = 6 from two independent experiments). Uninfected femurs (open squares) were measured for oxygen tension immediately following (n = 5) or 4 days after (n = 3) a mock inoculation procedure. Oxygen tension is reported as mmHg. Horizontal lines represent the mean. Error bars represent the SD. Dotted line represents the upper limit of detection.

Normal bone and bone marrow are intrinsically hypoxic, with a physiologic oxygen concentration range of 11.7 to 48.9 mmHg (1.5–6.4% O_2_), compared to atmospheric oxygen at approximately 160 mmHg (21% O_2_) [11,28]. TnSeq analysis demonstrated that the hypoxia-responsive SrrAB TCS is essential for *S*. *aureus* survival in bone, suggesting that bacterial invasion and the resulting inflammation associated with osteomyelitis trigger further decreases in skeletal oxygen availability. In order to determine the oxygen concentrations of *S*. *aureus* infected murine femurs during osteomyelitis, an Oxylite monitor was used to record oxygen tensions at the inoculation site at various times post-infection. In uninfected mice, average pO_2_ in the intramedullary canal was 45.2 mmHg, (**Fig 2**) consistent with previously reported bone marrow physoxia [28]. As infection progressed, the infectious focus became increasingly hypoxic, with an average oxygen tension of 14.3 mmHg at 10 days post-infection. This decreased oxygen tension was not due to the trauma induced by the inoculation procedure, as mock-infected bone showed an elevated mean pO_2_ of 77.5 mmHg by 4 days post-procedure (**Fig 2**). Collectively, these findings demonstrate that skeletal tissues become increasingly hypoxic during *S*. *aureus* osteomyelitis.

### The *srrAB* promoter is active in hypoxic skeletal tissues

**Fig 3:**
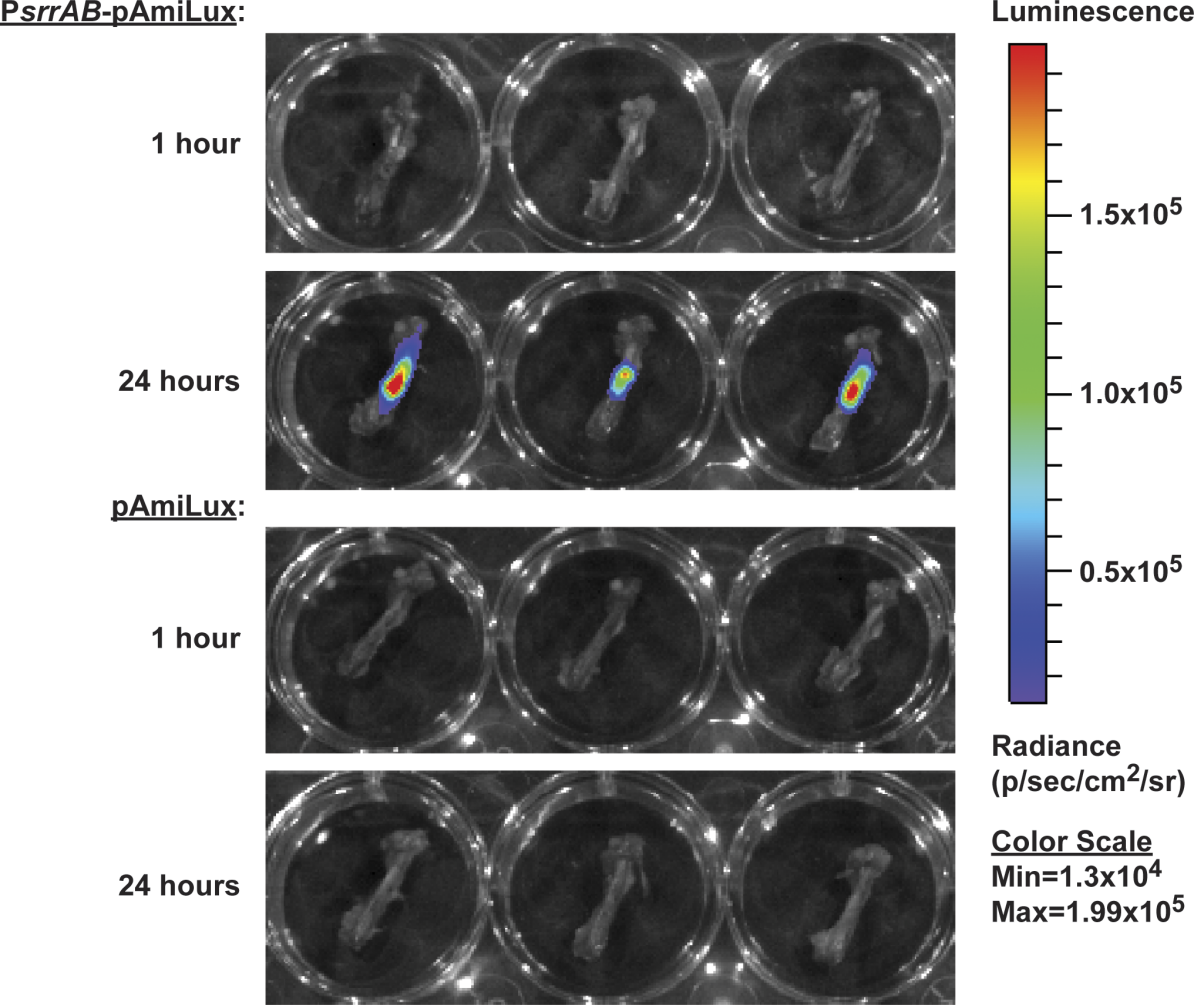
The *srrAB* promoter is active in hypoxic skeletal tissues. Groups of mice (n = 3 per group) were subjected to osteomyelitis by infection with WT bacteria containing either P*srrAB*-pAmiLux or pAmiLux (promoterless control). At 1 or 24 hours post-inoculation, infected femurs were explanted and immediately imaged on an IVIS 200 system (5 minute exposure).

Intravital pO_2_ monitoring revealed that skeletal tissues become increasingly hypoxic during osteomyelitis, with dramatically reduced oxygen tensions as early as 24 hours after infection. These data and the results of TnSeq analysis suggest that the *srrAB* promoter is active *in vivo*. To test the hypothesis that *srrAB* promoter activity increases with decreasing oxygen availability in infected skeletal tissues, a luminescent reporter construct was created in which expression of the *luxABCDE* operon is driven by the *srrAB* promoter. Mice were infected with WT *S*. *aureus* containing either this construct or a promoterless vector control, and at 1 hour or 24 hours post-infection infected femurs were explanted and immediately imaged for bioluminescence. No detectable luminescence above background was detected in femurs infected with WT bacteria containing the promoterless control plasmid at 1 hour or 24 hours after infection (**Fig 3**). In contrast, femurs infected with the P*srrAB*-pAmiLux construct showed no detectable luminescence above background at 1 hour post-infection, but displayed strong luminescent signal at 24 hours after infection, corresponding to the induction of hypoxia in infected skeletal tissues (**Fig 3**). Collectively, these results demonstrate that the *srrAB* promoter is activated *in vivo* during infection of hypoxic skeletal tissues.

### Neutrophil depletion rescues the intraosseous growth defect of an *srr* mutant

**Fig 4:**
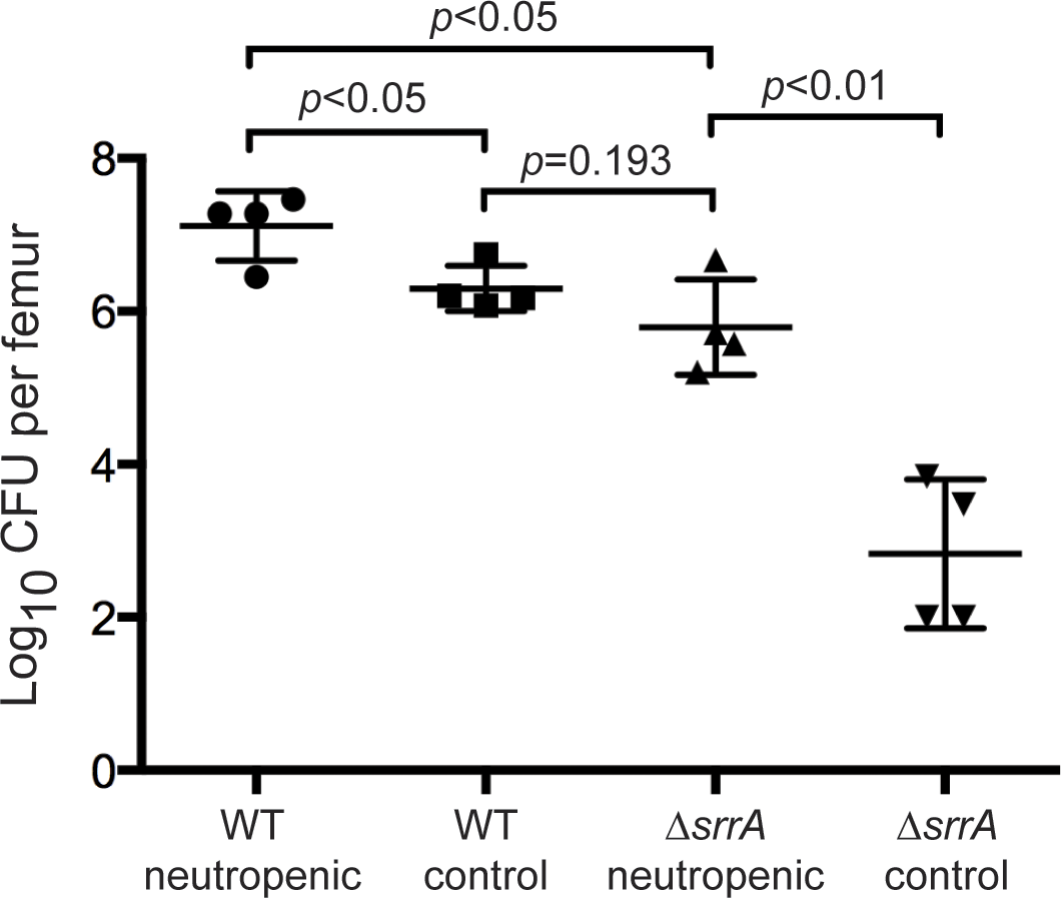
Neutrophil depletion rescues the intraosseous growth defect of an *srrA* mutant. Mice were given serial injections of anti-Ly6G monoclonal antibody. As a control, mice received injections of an isotype control antibody. At 14 days post-infection, femurs were processed for CFU enumeration. N = 4 mice per group. Horizontal lines represent the mean. Error bars represent SD. Significance determined by Students *t* test.

Intravital oxygen monitoring revealed hypoxia of skeletal tissues upon infection with *S*. *aureus*, suggesting that inflammation triggers a reduction in skeletal oxygen concentrations. Neutrophils are a significant source of both oxidative and nitrosative stresses *in vivo* and contribute to formation of oxygen-limited abscesses in response to staphylococcal infection [30]. To test the hypothesis that SrrAB is necessary to resist hypoxic and/or nitrosative stresses imposed by neutrophils *in vivo*, mice were either rendered neutropenic with serial anti-Ly6G (1A8) monoclonal antibody injections or given an isotype control monoclonal antibody and subsequently infected with WT or Δ*srrA* [31]. At 14 days post-infection, femurs were processed for enumeration of bacterial burdens. In mice treated with control antibody, a significant virulence defect was again observed in mice infected with the Δ*srrA* mutant (**Fig 4**). However, in mice administered the anti-Ly6G (1A8) antibody, a significant increase in bacterial burdens was observed upon infection with Δ*srrA*, such that bacterial burdens no longer differed significantly from non-neutropenic mice infected with WT (**Fig 4**). These results suggest that intraosseous survival requires SrrAB to resist hypoxic and/or nitrosative stresses produced by neutrophils in response to *S*. *aureus* osteomyelitis.

### *S*. *aureus* modulates quorum sensing and exotoxin production in response to oxygenation

The observation that *S*. *aureus* infection of murine skeletal tissues leads to dramatically reduced oxygen concentrations prompted further evaluations of how oxygenation impacts the production of staphylococcal virulence factors. We previously demonstrated that secreted toxins regulated by the accessory gene regulator (*agr*) locus are particularly important for the pathogenesis of *S*. *aureus* osteomyelitis [32,33]. The *agr* locus (*agrABCD*) encodes a quorum sensing system coupled to a TCS, and is responsible for growth phase-dependent regulation of a number of *S*. *aureus* virulence factors [34]. The response regulator of the *agr* locus, AgrA, directly regulates the production of alpha-type phenol soluble modulins (PSMs), which contribute significantly to the pathology of *S*. *aureus* osteomyelitis [32,35]. Indeed, alpha-type PSMs were found to be the sole mediators of cytotoxicity in concentrated culture supernatant towards murine and human osteoblasts *in vitro* [32]. However, a recent report demonstrated that alpha-type PSM expression is directly linked to alpha toxin (Hla) expression [36]. To verify that PSMs are the sole mediators of cytotoxicity toward osteoblastic cells in *S*. *aureus* concentrated supernatants, strain LAC*Δpsmα1–4* (Δ*psm*) containing the overexpression vector pOS1-*p*lgt driving *hla* expression in trans was tested for cytotoxicity towards osteoblastic cells (**S3 Fig**). While WT supernatant displayed maximum cytotoxicity, Δ*psm* and Δ*psm* pOS1-*p*lgt-*hla* did not show significantly different cytotoxicity from control. Deletion of *hla* in an erythromycin-resistant LAC background also failed to attenuate cytotoxicity (**S3 Fig**). Moreover, targeted inactivation of RNAIII in LAC did not decrease cytotoxicity, further supporting the AgrA-regulated alpha-type PSMs as the sole secreted mediators of cytotoxicity toward osteoblastic cells (**S4 Fig**).

**Fig 5:**
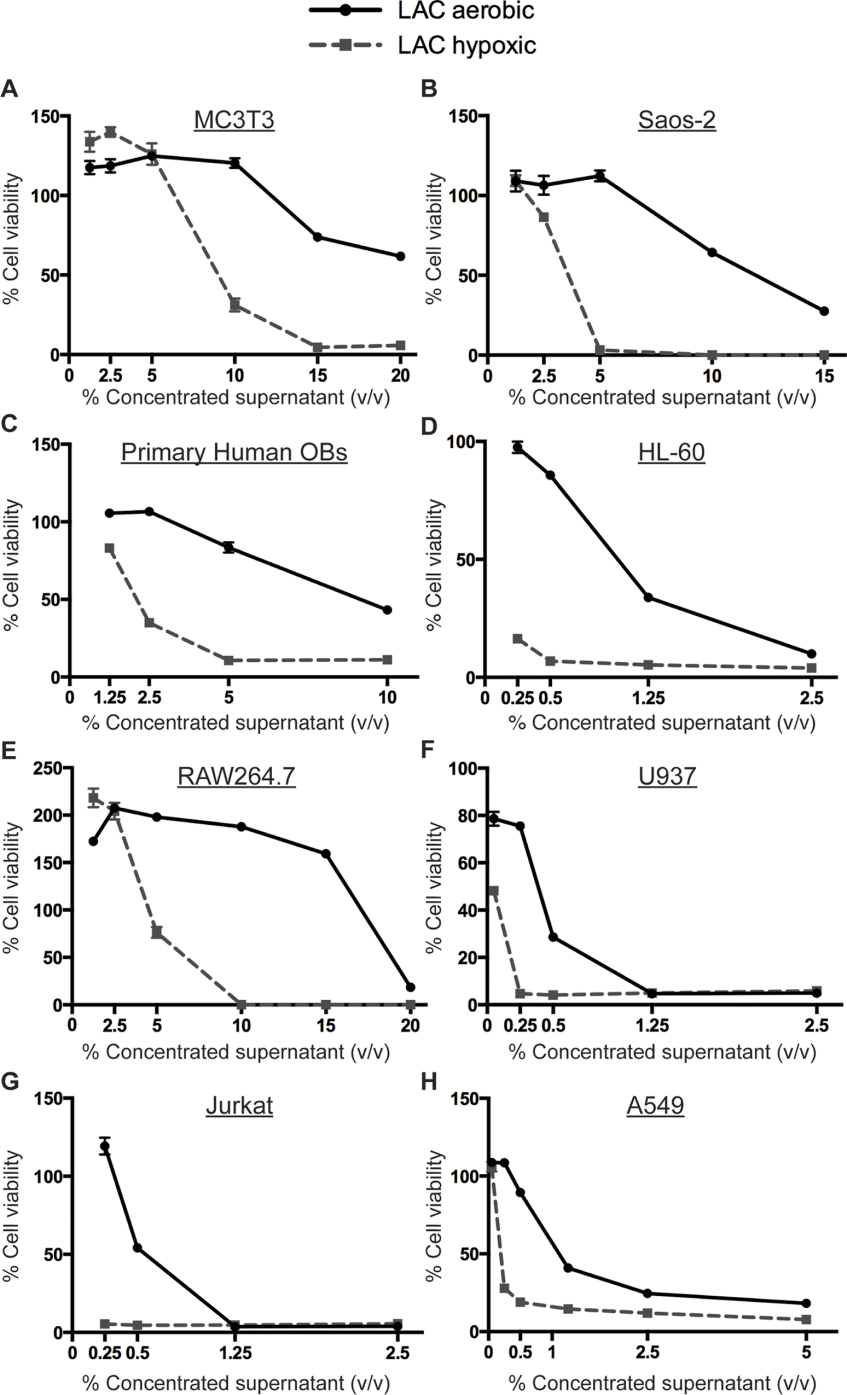
Hypoxically grown bacterial supernatants lead to increased cytotoxicity in human and murine cells. WT supernatants were prepared by inoculating 3 colonies into RPMI and 1% casamino acids (CA) and growing for 15 hours either aerobically or hypoxically. MC3T3 murine osteoblastic cells (A), Saos-2 human osteoblastic cells (B), primary human osteoblasts (C), HL-60 premyelocytes (D), RAW264.7 murine macrophages (E), U937 monocytic cells (F), Jurkat T cells (G), or A549 lung epithelial cells (H) were seeded into 96 well plates 24 hours prior to intoxication with concentrated supernatant or RPMI control. Cell viability was assessed 24 hours later. Results are expressed as percent of RPMI control (n = 10), and are representative of 2 biologic replicates with the exception of human primary osteoblasts, which represent a single experiment given the limited availability of this resource. Error bars represent the SEM.

To determine the impact of culture oxygenation on *S*. *aureus* exotoxin production, concentrated supernatants were prepared from *S*. *aureus* grown either aerobically or under limited oxygenation. Incubation of several different mammalian cell lines or primary human osteoblasts with varying amounts of concentrated culture supernatant demonstrated dose-dependent cytotoxicity that significantly increased if the bacteria were cultured under lower oxygenation (**Fig 5**). This phenomenon was not strain dependent, as hypoxic growth also increased the cytotoxicity of strains MW2 and Newman towards osteoblastic cells (**S5 Fig**). These results indicate that *S*. *aureus* virulence factor production is modulated in response to environmental oxygen levels.

**Fig 6:**
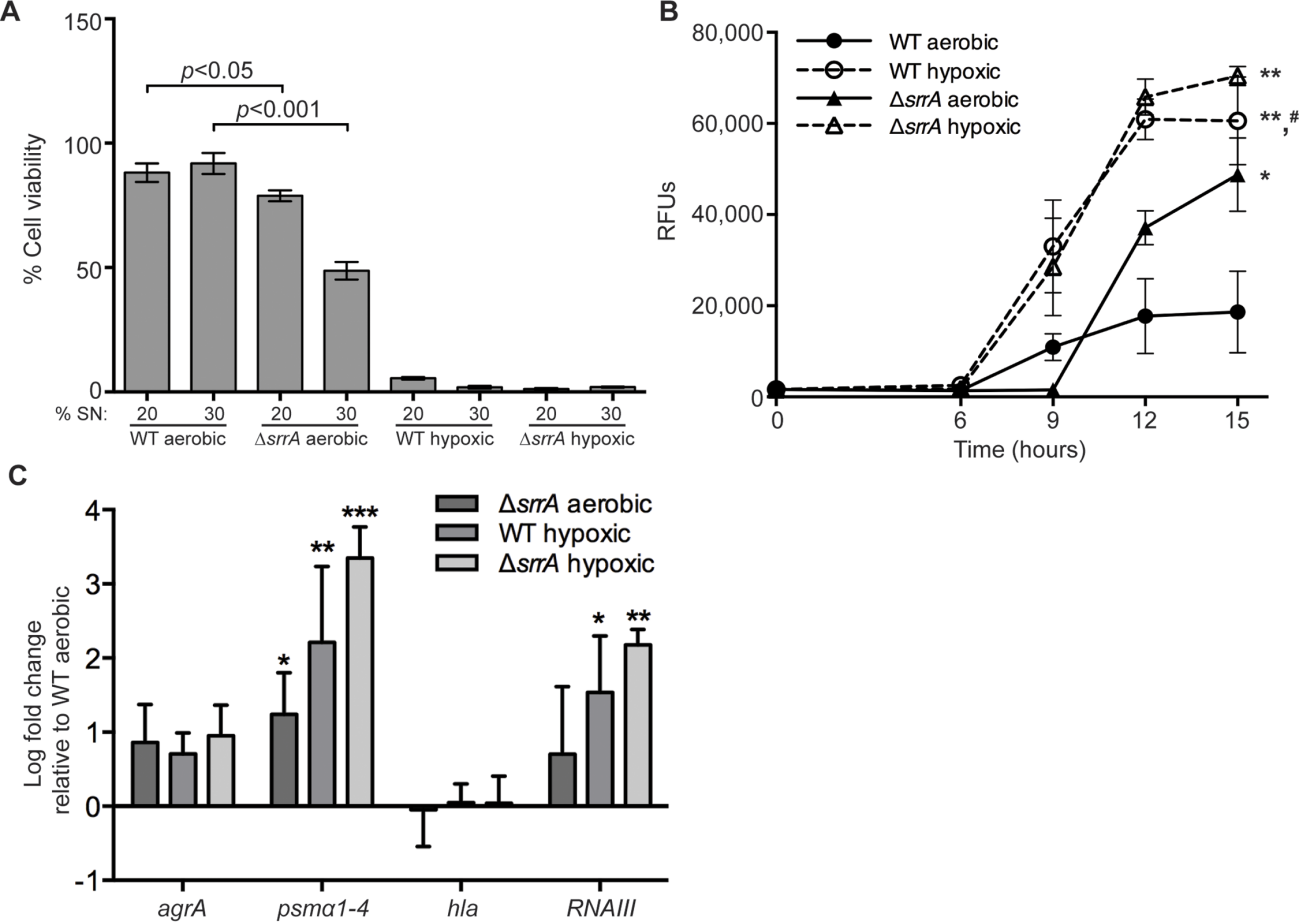
*S*. *aureus* modulates quorum sensing and exotoxin production in response to oxygenation. Supernatants from WT or Δ*srrA* were prepared by inoculating RPMI and 1% CA with a 1:1000 dilution from overnight cultures and growing for 15 hours either aerobically or hypoxically. Identical culture conditions were used to monitor quorum sensing and transcript levels (see below). (A) MC3T3 cells were seeded into 96 well plates at 5,000 cells per well. After 24 hours, growth media was replaced, and 20% or 30% of the total media volume was replaced with concentrated culture supernatant grown either aerobically or hypoxically, or an equivalent volume of RPMI. Cell viability was assessed 24 hours later, and results are expressed as percent of RPMI control (n = 10). Results are representative of at least three independent experiments. Error bars represent the SEM. (B) Agr-mediated quorum sensing was monitored using *agr*P3-dependent YFP expression in WT or Δ*srrA* strains grown aerobically or hypoxically as above. YFP relative fluorescent units (RFUs) were averaged from 3 technical replicates. Error bars represent the SD. Data shown are an average of 3 biologically independent experiments. RFUs monitored at 0, 6, 9, 12, and 15 hours after back-dilution from overnight culture. * and ** represent *p*<0.05 and 0.01, respectively relative to WT aerobic at 15 hours as determined by Student’s *t* test. # represents *p*<0.05 relative to Δ*srrA* aerobic. (C) cDNA samples from WT or Δ*srrA* strains grown aerobically or hypoxically as above for 15 hours were subjected to qRT-PCR. Graph depicts fold change of the indicated transcripts relative to WT aerobic transcript level. Data shown are an average of 3 biologically independent experiments. Error bars represent the SEM. Significance was determined by two way ANOVA. * denotes *p*<0.05, ** denotes *p*<0.01, and *** denotes *p*<0.001 relative to WT aerobic.

SrrAB regulates select virulence factors under microaerobic conditions in part by directly interacting with the *agr* P2 and P3 promoters [27,29]. This observation, combined with the role of SrrAB in responding to hypoxic stresses led to the hypothesis that SrrAB may regulate quorum sensing and virulence factor production in response to changes in oxygenation. To investigate the impact of SrrAB on PSM-mediated killing of osteoblasts, osteoblastic cells were incubated with varying amounts of culture supernatant from WT or Δ*srrA* strains grown either aerobically or under hypoxia. Aerobically grown Δ*srrA* supernatants demonstrated dose-dependent killing of murine osteoblasts that was significantly increased compared to aerobically grown WT supernatants, mimicking the effect of hypoxia on cytotoxicity (**Fig 6A**). The cytotoxicity of aerobically grown Δ*srrA* was diminished by expression of the *srrAB* locus in trans (**S6 Fig**). These data suggest that SrrAB represses PSM-mediated cytotoxicity under aerobic conditions.

Because SrrAB repressed PSM-mediated cytotoxicity under aerobic conditions, we hypothesized that SrrAB impacts quorum sensing in response to oxygenation. To test this hypothesis, the reporter plasmid pDB59 (*agr*P3 promoter driving YFP expression) was introduced into WT and Δ*srrA* [37]. Aerobically grown WT cultures demonstrated significantly lower *agr*P3 activation compared to cultures grown under limited oxygenation (**Fig 6B**). This decrease was partially SrrAB dependent, as aerobically grown Δ*srrA* strains demonstrate a 2-fold higher expression of *agr*P3 than aerobic WT cultures (**Fig 6B**). To further confirm these results, quantitative RT-PCR was conducted on aerobically and hypoxically grown cultures of WT and Δ*srrA*. Transcription of *agrA* was increased relative to aerobically grown WT for both Δ*srrAB* and hypoxically grown cultures. Hypoxically grown cultures also demonstrated significantly elevated levels of *psmα* and *RNAIII transcripts* (**Fig 6C**). Inactivation of SrrAB resulted in an over 30-fold increase in *psmα1–4* transcription and a near 20-fold increase in *RNAIII* expression under aerobic conditions. Under hypoxic conditions, inactivation of *srrAB* resulted in a 3000-fold and 160-fold increase in *psmα1–4* and *RNAIII* transcript levels, respectively. Collectively, these data indicate that *S*. *aureus* quorum sensing and resultant cytotoxicity towards mammalian cells is modulated in an SrrAB-dependent manner in response to changing oxygen availability, and further define SrrAB as an important regulator of metabolic and virulence adaptations during invasive infection.

## Discussion

TnSeq analysis during experimental osteomyelitis revealed *S*. *aureus* genes essential for invasive infection. Among the mutants with reduced *in vivo* fitness was one TCS, SrrAB, which was previously characterized as a coordinator of hypoxic and nitrosative stress responses [26]. SrrAB was originally identified as a regulator of oxygen-dependent toxic shock syndrome toxin-1 (TSST-1) expression, and was noted to have homology to the global respiratory regulator ResDE in *Bacillus subtilis* [29,38]. Subsequent analyses revealed that SrrA is capable of binding to the *agr* P2 and P3 promoter regions, and that overexpression of SrrAB reduces virulence in a rabbit endocarditis model [27]. These findings indicate a link between SrrAB and quorum sensing and suggest that oxygenation could impact staphylococcal virulence. Yet the specific signal(s) that activate SrrAB, and the mechanism by which this system modulates quorum sensing have yet to be determined.

Our data suggest that aerobic growth of *S*. *aureus* limits quorum sensing and *agr*-dependent virulence factor production in a manner that is partially dependent on SrrAB. Conversely, hypoxic growth results in significantly increased cytotoxicity toward mammalian cells. Since equivalent bacterial densities were achieved under conditions of hypoxic and aerobic growth, these results imply that the output of quorum sensing can be functionally uncoupled from bacterial density by changes in culture oxygenation. Such uncoupling could be particularly advantageous for quenching of virulence factor production in environments with higher oxygen availability, such as during colonization of the skin or nares. Since inactivation of SrrAB under aerobic conditions failed to fully restore quorum sensing and cytotoxicity to the levels observed with hypoxic growth, it is likely that this phenomenon is a result of multiple factors. Additional studies are therefore needed to determine the SrrAB-dependent and SrrAB–independent mechanisms by which oxygenation regulates quorum sensing. To this end, it has previously been demonstrated that both the *S*. *aureus* autoinducing peptide (AIP) and AgrA can be functionally inactivated by oxidation [39,40], suggesting a potential SrrAB-independent mechanism for modulation of quorum sensing by oxygen. Moreover, the *S*. *aureus* genome is known to encode other redox-sensitive regulators such as Rex, MgrA, SarA, and AirSR [41–45]. It is therefore possible that environmental oxygen is not a direct regulator of quorum sensing, but rather that a change in the redox status of the bacterial cell or oxidative damage triggers changes in virulence factor production. Nevertheless, our findings suggest that shifts in available oxygen, as well as the inherent differences in physiologic oxygen concentrations in various host tissues, could have a significant impact on staphylococcal virulence. Additionally, these data highlight the importance of *in vitro* culture conditions on the study of staphylococcal virulence.

Global transcriptional analyses defined the SrrAB regulon of *S*. *aureus* under conditions of aerobic and hypoxic growth. Interestingly, although some overlap was noted with the previously reported SrrAB nitrosative stress regulon, we identified additional SrrAB-regulated genes under conditions of changing oxygenation [26]. Although these findings may relate to technical issues or strain-dependent differences in gene regulation, they suggest that SrrAB may integrate multiple environmental signals, or that oxidative and nitrosative stress trigger a common endogenous bacterial pathway that activates SrrAB. In order to begin defining the host components that trigger hypoxic or nitrosative stress responses in *S*. *aureus*, we examined the role of neutrophils during osteomyelitis. Neutrophils impose nitrosative and oxidative stress to invading pathogens through the respiratory burst, which generates reactive oxygen and nitrogen species. Moreover, neutrophils contribute to tissue hypoxia through abscess formation [30]. In support of the role of SrrAB in resisting such hypoxic and nitrosative stresses, we found that neutrophil depletion partially rescued the virulence defect of an *srrA* mutant. Additional studies are needed to parse out the effects of neutrophil-derived reactive oxygen and nitrogen species versus abscess-associated tissue hypoxia on the survival of *S*. *aureus*. Furthermore, it is likely that other innate and adaptive immune responses contribute to changes in tissue oxygenation and thus the redox status of invading pathogens.

In addition to the genes encoding SrrAB and its targets, TnSeq analysis during osteomyelitis revealed a large number of staphylococcal mutants with compromised fitness *in vivo*. Many of the genes identified as essential or compromised during *in vivo* growth can be broadly classified as associated with metabolism. In contrast, very few prototypical virulence factors were identified as essential for osteomyelitis. The lack of traditional secreted virulence factors identified through TnSeq analysis is not surprising due to the nature of the technique. Infection with a pooled transposon library allows for mutants deficient in a particular gene to potentially co-opt bacterial factors from other mutants. Indeed, this phenomenon has been characterized for the exchange of metabolic intermediates in *S*. *aureus*, and it is conceivable that secreted virulence factors could be “shared” in a similar manner [33]. An additional limitation of TnSeq analysis is the requirement for a short outgrowth step in liquid media following harvest of the transposon library from infected bone. This outgrowth step could potentially confound our results by altering the fitness of mutants recovered from infected tissues. However, a brief outgrowth step is necessary to decrease the amount of murine DNA present in the femur homogenate and allow for effective sequencing of bacterial DNA, and is a common adjustment in TnSeq analyses of infected tissues [5, 8, 22].

TnSeq analysis of staphylococcal osteomyelitis paralleled a previous TnSeq analysis of staphylococcal growth in soft tissue abscesses [8]. In fact, 40 of the 65 genes identified as essential for growth during osteomyelitis were also essential for growth in murine abscesses. This observation is consistent with our previous data showing that osteomyelitis is characterized by exuberant abscess formation in the bone marrow [32], and suggests common stresses are encountered by staphylococci in neutrophil-rich inflammatory lesions. However, 25 of the 65 genes essential for intraosseous survival were not found to be essential for abscess growth, and may reflect unique adaptations to colonization of skeletal tissues.

Of the genes required for *S*. *aureus* survival during invasive infection, many encode hypothetical proteins or proteins without a previously characterized role in virulence. This observation highlights the power of TnSeq analysis as an unbiased evaluation of the genetic requirements for bacterial survival in host tissues. In summary, the results of this study elucidate bacterial survival strategies during invasive infection, link changes in environmental oxygen to staphylococcal quorum sensing and virulence, and provide a firm foundation to identify new targets for antimicrobial and vaccine design.

## Materials and Methods

### Ethics statement

All experiments involving animals were reviewed and approved by the Institutional Animal Care and Use Committee of Vanderbilt University and performed according to NIH guidelines, the Animal Welfare Act, and US Federal law.

### Bacterial strains and growth conditions

The *S*. *aureus* TnSeq library in strain HG003 has been previously described [8]. All other experiments were conducted in an erythromycin-sensitive, tetracycline-sensitive derivative of the USA300 strain LAC (AH1263), which served as the wildtype (WT) unless otherwise noted [46]. Strain LACΔ*psmα1–4* has been previously described [32,47]. Strains Δ*srrA*, Δ*qoxA*, Δ*qoxC*, Δ*pflA*, and Δ*pflB* in the LAC background were created by bacteriophage phi-85-mediated transduction of *erm*-disrupted alleles from the respective JE2 strain mutants obtained from the NARSA transposon library [48]. Strains Δ*psm* pOS1-*p*lgt and Δ*psm* pOS1-*p*lgt-*hla* were provided by Dr. Juliane Bubeck-Wardenburg [36]. Construction of strain LAC Δ*RNAIII* is described below. Plasmid pDB59 (*agr*-P3-YFP) was electroporated into LAC or Δ*srrA* for monitoring of *agr*-dependent quorum sensing [37]. All strains were grown in glass Erlenmeyer flasks at 37°C with orbital shaking at 180 rpm. All S. *aureus* strains were grown in Tryptic Soy Broth (TSB), Brain-Heart Infusion (BHI), or Roswell Park Memorial Institute medium (RPMI) supplemented with 1% casamino acids (CA). *Escherichia coli* was grown in Luria Broth (LB). Erythromycin and chloramphenicol were added to cultures at 10 μg ml^-1^ where indicated. Ampicillin was added to cultures at 100 μg ml^-1^ where indicated. Cadmium chloride was added to cultures at 0.1 mM where indicated. A 5:1 flask to volume ratio was utilized unless otherwise noted. For comparative growth analyses, overnight aerobic cultures were back-diluted 1:1000 into fresh TSB or BHI media and optical density at 600 nm (OD_600_) was monitored over time.

### Construction of LAC Δ*RNAIII*

*RNAIII* including upstream and downstream flanking regions were amplified using primers 5'-GCATGCGTCGATATCGTAGCTGGGTCAG-3' and 5'-GAATTCGAAGTCACAAGTACTATAAGCTGCG-3', and cloned into the *Hinc*II site of pUC18 [49] to create pGAW1. To delete RNAIII, inverse PCR was performed with primers 5'-TTTGGGCCCTATATTAAAACATGCTAAAAG-3' and 5'-TTTCTCGAGGTAATGAAGAAGGGATGAGTT-3' amplifying RNAIII flanking regions and the remaining plasmid backbone of pGAW1. The vector was religated after treatment with Polynucleotide Kinase (New England Biolabs, MA) and designated pGAW3. To insert an antibiotic resistance cassette, pGAW3 was digested with *Apa*I and *Xho*I, religated with the *Apa*I*-Xho*I fragment from pJC1075 [50] (*cadCA*, conferring resistance to cadmium) and designated pGAW6. The *Sph*I*- Kpn*I fragment from pGAW6 was cloned into the allelic replacement vector pJC1202 [50] using the same restriction sites and designated pGAW7. Strain RN4220 was electroporated with plasmid pGAW7 and plated on GL agar containing 5 μg chloramphenicol ml^–1^ at 30°C. Allelic exchange was carried out as previously described [50]. Phage 80a was then used to transduce the mutation into LAC to generate LAC *RNAIII*::*cad*, herein designated LAC Δ*RNAIII*.

### Construction of an SrrAB overexpression plasmid

To express *srrAB* in trans, the *srrAB* open reading frame was PCR amplified from genomic DNA of LAC using primers 5’-ATCTCGAGATGTCGAACGAAATACTTATCG-3’ and 5’- ATGGATCCTTCAATTTTATTCTGGTTTTGGTAG-3’. The resulting *srrAB* amplicon was then cloned into the shuttle vector pOS1 under control of the *lgt* promoter [51]. As a control, wild type and Δ*srrA* strain LAC were transformed with pOS1-*lgt* lacking an insert.

### Construction of an *srrAB* bioluminescent reporter

To examine expression of *srrAB in vivo* the *srrAB* promoter was PCR amplified from genomic DNA of LAC using primers 5’-TACCCGGGTGTATTTATCACAAAGTTTGAGAAT-3’ and 5’-ATCGTCGACACAGGTCATACCTCCCAC-3’. The resulting amplicon was then cloned into the shuttle vector pAmiLux, kindly provided by Dr. Julian Davies [52]. As a control, wild type stain LAC was transformed with pAmilux lacking an insert.

### Murine model of osteomyelitis and micro-computed tomographic analysis

Osteomyelitis was induced in 7- to 8-week old female C57BL/6J mice as previously reported [32]. An inoculum of 1x10^6^ colony-forming units (CFU) in 2 μl PBS was delivered into murine femurs. For some experiments, mice were rendered neutropenic by serial intraperitoneal injections of an anti-Ly6G (clone 1A8) monoclonal antibody (BioXcell, West Lebanon, NH) at days -3, 0, 4, 7, and 10 post-infection. As a control, mice received serial injections of an isotype control antibody (rat IgG2a). At various times post-infection, mice were euthanized and the infected femur was removed and either processed for CFU enumeration or imaged by micro-computed tomography (microCT). For CFU enumeration, femurs were homogenized and plated at limiting dilution on Tryptic Soy Agar (TSA). Analysis of cortical bone destruction was determined by microCT imaging as previously described [32]. Differences in cortical bone destruction and bacterial burdens were analyzed using Student’s *t* test.

### Bioluminescent imaging

Bioluminescent imaging was performed on infected femurs explanted into sterile multiwell plates at either 1 or 24 hours after infection with WT bacteria containing P*srrAB*-pAmiLux or pAmiLux. Luminescence was measured in an IVIS 200 Imaging System (Perkin Elmer, Akron, OH) with an exposure time of 5 minutes, f-stop of 1, and binning of 4. All images were manually scaled to the same minimum and maximum values to exclude background and include the peak luminescent value.

### Intravital measurements of oxygen concentration

Intravital oxygen concentrations were measured in infected femurs using an Oxylite (Oxford Optronix, United Kingdom) oxygen and temperature monitor in conjunction with a flexible bare-fibre sensor. Mice were anesthetized with isoflurane and the surgical incision was re-opened. Oxygen readings were obtained by insertion of the sensor directly through the intramedullary canal and into the infectious focus. Measurements from the probe were recorded at least 5 minutes after probe placement to allow for temperature equilibration and stabilization of oxygen readings.

### Transposon sequencing analysis of experimental acute osteomyelitis

The *S*. *aureus* TnSeq library in the HG003 background has been previously described [8]. In order to identify potential bottlenecks in the murine osteomyelitis model that could confound TnSeq analysis, groups of mice were first infected with strain HG003 using an inoculum of 5x10^6^ CFU and then at various times post-infection the infected femurs were collected and processed for CFU enumeration. Day 5 was chosen as a timepoint for TnSeq analysis of acute osteomyelitis as it likely represents the first bottleneck encountered by invading bacteria. To prepare the TnSeq library for inoculation into murine femurs, an aliquot of the library containing 5x10^7^ CFU/ml was thawed and inoculated into 100 ml of BHI media in a 500 ml Erlenmeyer flask. This culture was incubated at 37°C for 12 hours and then back-diluted 1:100 into fresh BHI at the same flask to volume ratio and grown an additional 3 hours. Bacterial cells were harvested by centrifugation and resuspended in PBS to a concentration of 7x10^6^ CFU in 2 μl PBS. This inoculum dose failed to cause mortality or severe morbidity requiring euthanasia when administered to five wildtype mice by retro-orbital injection (mice were monitored for a total of 4 days). Genomic DNA was prepared from the inoculum using a Qiagen DNeasy Kit with 40 μg ml^-1^ lysostaphin added to the lysis buffer. The inoculum was used to initiate experimental osteomyelitis in groups of mice as above. Another equivalent aliquot of the inoculum was seeded into a 50 ml BHI culture in a 250 ml Erlenmeyer flask. This culture was grown for 24 hours, after which time the bacterial cells were harvested and genomic DNA was prepared as above. This genomic DNA served as the *in vitro* comparator for TnSeq analysis. At 5 days post-infection, mice inoculated with the TnSeq library were euthanized, and the infected femurs were harvested and homogenized in 1 ml of PBS. 500 μl of this homogenate was archived by freezing at -80°C in 20% glycerol and the remaining 500 μl of the homogenate was seeded into 4ml of BHI media and cultured at 37°C and 180 rpm shaking for 5.5 hours. Bacteria were then collected by centrifugation and subjected to genomic DNA preparation as above. Recovered bacteria from 2 mice were pooled, and 3 biologically independent groups of mice were analyzed separately. Genomic DNA samples were subsequently prepared for sequencing on an Illumina HiSeq 2000 (Tufts University Genomic Core Facility). Sequencing, data analysis, and fitness calculations were performed as previously reported [8]. Briefly, a “dval” was calculated for each gene in each condition (inoculum, *in vitro* comparator, or osteomyelitis). The dval represents the observed number of mappable reads of insertions in a gene, divided by the number of mappable reads of insertions predicted for that gene based on its size relative to the genome and the total number of mappable reads obtained for that experiment. Genes with dval of ≤0.01 were considered “essential” in a given condition. Genes with dval of >0.01 but ≤0.1 were considered “compromised” in a given condition, whereas genes with dval >0.1 were considered “fit”. A dval ratio was calculated by dividing the dval of a given gene in osteomyelitis by the dval of the same gene during *in vitro* comparator growth.

### RNA isolation and Genechip analysis

For genechip analysis, aerobic cultures of WT or Δ*srrA* were prepared as follows. Three colonies of WT or Δ*srrA* were inoculated into 10 ml of TSB in a 50 ml Erlenmeyer flask. This culture was grown overnight then back-diluted 1:1000 into 50 ml of TSB in a 250 ml flask. The back-diluted cultures were grown at 37°C and 180 rpm orbital shaking until OD_600_ reached 0.5, at which time an equal volume of ice-cold 1:1 acetone:ethanol was added and the cultures were stored at -80°C until processed for RNA isolation. For comparison of RNA from aerobic versus hypoxic conditions, TSB cultures of WT or Δ*srrA* were incubated overnight as above, back-diluted 1:1000 into 100 ml of TSB in a 500 ml flask and grown to an OD_600_ of 0.5. Fifty milliliters of the culture was then placed into a tightly capped 50ml conical (hypoxic condition) and incubated for one hour at 37°C before mixture with acetone:ethanol and storage at -80°C. The remaining 50 ml of culture was moved to a 250 ml Erlenmeyer flask (aerobic condition) and incubated for one hour at 37°C before mixture with acetone:ethanol and storage at -80°C. For RNA isolation, bacterial cells were harvested by centrifugation and resuspended in LETS buffer (0.1 M LiCl, 10 mM EDTA, 10 mM Tris HCl, 1% SDS). The resuspended cells were disrupted in the presence of 0.5 mm RNAase-free zirconium oxide beads in a Bullet Blender (Next Advance, Averill Park, NY, USA). Disrupted cells were heated at 55°C for 5.5 minutes and centrifuged for 10 minutes at 15,000 rpm. The upper phase was collected and transferred to a new tube before adding 1 ml of TRI-Reagent. After mixing, 200 μl of chloroform was added, and the resultant solution was mixed vigorously for 15 seconds. Samples were centrifuged at 15,000 rpm for 10 min, and the aqueous phase was transferred to a new tube. RNA was precipitated with isopropyl alcohol and subsequently washed with 70% ethanol before drying and resuspension in deionized water. RNA samples were subsequently treated with DNase I and re-purified with a GeneJET RNA Cleanup Kit (Thermo Fisher Scientific, Waltham, MA, USA).

For Genechip analysis, RNA samples were labeled, hybridized to commercially available *S*. *aureus* Affymetrix Genechips, and processed as per the manufacturer’s instructions (Affymetrix, Santa Clara, CA, USA). Briefly, 10 μg of each RNA sample was reverse transcribed, resulting cDNA was purified using QIAquick PCR Purification Kits (Qiagen, Germantown, MD, USA), fragmented with DNase I (Ambion, Carlsbad, CA, USA), and 3’ biotinylated using Enzo Bioarray Terminal Labeling Kits (Enzo Life Sciences, Farmingdale, NY, USA). A total of 1.5 μg of a labeled cDNA sample was hybridized to a *S*. *aureus* GeneChip for 16 hr at 45°C, processed, and scanned in an Affymetrix GeneChip 3000 7G scanner as previously described [53,54]. Signal intensity values for each GeneChip qualifier were normalized to the average signal of the microarray to reduce sample labeling and technical variability and the signal for the biological replicates were averaged using GeneSpring GX software (Agilent Technologies, Redwood City, CA, USA) [54–57]. Differentially expressed transcripts were identified as RNA species that generated a two-fold increase or decrease in WT cells in comparison to Δ*srrA* cells during aerobic and hypoxic conditions (*t*-test, *p* = 0.05). All related GeneChip data files were deposited in the NCBI Gene Expression Omnibus repository in the MIAME-compliant format.

### Supernatant preparations

*S*. *aureus* strains were used to inoculate RPMI + 1% CA in glass Erlenmeyer flasks. For aerobic growth, the flask opening was covered lightly with aluminum foil. For hypoxic growth, the flask opening was sealed with a rubber stopper. Cultures were grown for 15 hours. Supernatants were collected after culture centrifugation, and were subsequently filtered through a 0.22 μm filter and concentrated with an Amicon Ultra 3 kDa nominal molecular weight limit centrifugal filter unit (Millipore, Billerica, MA, USA) per the manufacturer’s instructions. Following concentration, supernatants were filter sterilized again and frozen at -80°C until used.

### Mammalian cell culture and cytotoxicity assays

Primary human osteoblasts were obtained from Lonza (Basel, Switzerland) and cultured per manufacturer’s recommendations. All cell lines were obtained from the American Type Culture Collection (ATCC) and propagated at 37°C and 5% CO_2_ according to ATCC recommendations. Media was replaced every 2–3 days. All cell culture media was prepared with 1X penicillin/streptomycin and filter sterilized using a 0.22 μm filter prior to use. MC3T3 E-1 cells were cultured in α-MEM, supplemented with 10% fetal bovine serum (FBS). The RAW264.7, Saos-2, and A549 cell lines were grown in Dulbecco’s MEM (DMEM) with 10% FBS, McCoy’s 5A medium with 15% FBS, and F-12K medium with 10% FBS, respectively. The Jurkat, U937, and HL-60 cell lines were propagated using RPMI with 10% FBS. Cytotoxicity assays were performed in 96-well tissue culture grade plates. Cells were seeded one day prior to intoxication with *S*. *aureus* concentrated supernatants or sterile RPMI diluted in the recommended cell culture medium. The following cell densities were used for cytotoxicity assays: MC3T3 E1 murine pre-osteoblastic cells at 5,000 cells per well, primary human osteoblasts at 3,500 cells per well, Saos-2 human osteoblastic cells at 10,000 cells per well, RAW264.7 murine macrophage cells at 10,000 cells per well, A549 lung epithelial cells at 5,000 cells per well, U937 monocytic cells at 15,000 cells per well, HL-60 premyelocytes at 20,000 cells per well, and Jurkat T cells at 50,000 cells per well. Concentrated supernatants were added as dilutions, by mixing between 0.1 μl to 60 μl in a total volume of 200 μl per well to give a dilution spectrum of 0.05%-30% concentrated supernatant (volume/volume). Cell lines in suspension were centrifuged at 3000 x g for 5 minutes prior to intoxication. Cell viability was assessed with CellTiter AQueous One (Promega, Madison, WI, USA) per the manufacturer’s instructions at 24 hours post-intoxication.

### YFP fluorescence measurements

For fluorescence analysis, overnight cultures of WT and Δ*srrA* containing the pDB59 reporter plasmid were back-diluted 1:1000 into 10 ml of RPMI + 1% CA with chloramphenicol in 50ml Erlenmeyer flasks and grown either aerobically or hypoxically as above. YFP was measured using an excitation of 485/20 and emission of 528/20 in a BioTek Synergy HT 96-well plate reader at 0, 6, 9, 12, and 15 hours after back-dilution.

### Quantitative RT-PCR

Bacteria were grown for 15 hours as for YFP fluorescence measurements, mixed with 1:1 acetone:ethanol, and stored at -80°C until processed for RNA isolation. RNA isolation was performed as for Genechip analysis. Reverse transcription using 2 μg of RNA and M-MLV reverse transcriptase (Promega, Madison, WI, USA) was performed following the manufacturer’s instructions. Quantitative RT-PCR (qRT-PCR) was performed using iQ SYBR Green Supermix (Bio-Rad, Hercules, Ca, USA) and the cDNA generated above for each primer pair, including a no reverse transcriptase negative control for 16S rRNA. PCR was conducted on a CFX96 qPCR cycler (Bio-Rad, Hercules, Ca, USA). The cycling program was carried out as recommended by the manufacturer with an annealing temperature of 56°C. Fold-changes were calculated from Ct values averaged from three technical replicates for at least three biological replicates after normalizing to 16S rRNA. The qRT-PCR primer sequences for *agrA*, *hla*, and *RNAIII* were previously published [58]. The qRT-PCR primer sequence for 16S rRNA was also previously published [36].

## Supporting Information

S1 Table Genes identified as essential for osteomyelitis by TnSeq analysis. (PDF)

S2 Table Transposon mutants with compromised fitness during osteomyelitis, but not *in vitro* growth, as identified by TnSeq analysis. (PDF)

S3 Table Transcripts differentially regulated by SrrAB during aerobic growth. (PDF)

S4 Table Transcripts differentially regulated by SrrAB during hypoxic growth. (PDF)

S1 Fig Evaluation of HG003 growth kinetics during experimental osteomyelitis. Groups of mice were subjected to osteomyelitis using strain HG003. Infected femurs were harvested at 1, 3, 5, 7, and 12 days post-infection and processed for CFU enumeration (n = 3). Horizontal lines represent the mean. Error bars represent the SD. Significance was determined by Students *t* test.
(TIF)

S2 Fig Growth kinetics of *ΔsrrA* and select SrrAB-regulated mutants under aerobic or hypoxic conditions. Growth of WT, *ΔsrrA*, *ΔpflA*, *ΔpflB*, *ΔqoxA*, and *ΔqoxC* strains was monitored by OD_600_ with 3 technical replicates at 0, 2, 4, 6, 8, and 24 hours. Data shown is representative of 3 biologically independent experiments. Error bars represent the SEM. (A) Strains grown aerobically in BHI, which served as the *in vitro* comparator media during TnSeq analysis. (B) Strains grown aerobically in TSB. (C) Strains grown hypoxically in TSB by tightly capping Erlenmeyer flasks.
(TIF)

S3 Fig Alpha-hemolysin does not impact cytotoxicity of concentrated *S*. *aureus* supernatants towards osteoblastic cells. Saos-2 osteoblastic cells were seeded into 96-well plates and cell viability was assessed 24 hours after intoxication with supernatant (30% total media volume) from the indicated strains following hypoxic growth. Results are expressed as percent of RPMI control (n = 10). Error bars represent the SEM. LAC^R^ indicates an erythromycin-resistant derivative of LAC used for construction of the *hla* mutant.
(TIF)

S4 Fig Inactivation of RNAIII does not impact cytotoxicity of concentrated *S*. *aureus* supernatants towards osteoblastic cells. MC3T3 osteoblastic cells were seeded into 96-well plates and cell viability was assessed 24 hours after intoxication with supernatant (30% total media volume) from the indicated strains following hypoxic growth. Results are expressed as percent of RPMI control (n = 10). Error bars represent the SEM.
(TIF)

S5 Fig Hypoxic growth enhances the cytotoxicity of strains MW2 and Newman. WT supernatants were prepared from strains MW2 (A) and Newman (B) by inoculating 3 colonies into RPMI and 1% casamino acids (CA) and growing for 15 hours either aerobically or hypoxically. MC3T3 murine osteoblastic cells were seeded into 96 well plates 24 hours prior to intoxication with concentrated supernatant or RPMI control. Cell viability was assessed 24 hours later. Results are expressed as percent of RPMI control (n = 10), and are the average of 2 biologic replicates. Error bars represent the SEM.
(TIF)

S6 Fig Expression of SrrAB in trans decreases cytotoxicity of aerobic cultures. MC3T3 cells were intoxicated with 30% total media volume of RPMI control or concentrated supernatant from the indicated strains after aerobic or hypoxic growth. Cell viability was determined 24 hours after intoxication. Results are expressed as percent of RPMI control (n = 10). Error bars represent the SEM. Significance was determined by Students *t* test.
(TIF)

## References

[1] Kuehnert MJ, Kruszon-Moran D, Hill HA, McQuillan G, McAllister SK, et al (2006) Prevalence of *Staphylococcus aureus* nasal colonization in the United States, 2001–2002. J Infect Dis 193: 172–179. 16362880 10.1086/499632

[2] van Opijnen T, Bodi KL, Camilli A (2009) Tn-seq: high-throughput parallel sequencing for fitness and genetic interaction studies in microorganisms. Nat Methods 6: 767–772. 10.1038/nmeth.1377 19767758 PMC2957483

[3] Barquist L, Boinett CJ, Cain AK (2013) Approaches to querying bacterial genomes with transposon-insertion sequencing. RNA Biol 10: 1161–1169. 10.4161/rna.24765 23635712 PMC3849164

[4] Roux D, Danilchanka O, Guillard T, Cattoir V, Aschard H, et al (2015 ) Fitness cost of antibiotic susceptibility during bacterial infection. Sci Transl Med 7: 297ra114 10.1126/scitranslmed.aab1621 26203082

[5] Bachman MA, Breen P, Deornellas V, Mu Q, Zhao L, et al (2015) Genome-Wide Identification of *Klebsiella pneumoniae* Fitness Genes during Lung Infection. MBio 6. 10.1128/mBio.00775-15 PMC4462621 26060277

[6] Shan Y, Lazinski D, Rowe S, Camilli A, Lewis K (2015) Genetic basis of persister tolerance to aminoglycosides in *Escherichia coli* . MBio 6. 10.1128/mBio.00078-15 PMC4453570 25852159

[7] Turner KH, Wessel AK, Palmer GC, Murray JL, Whiteley M (2015) Essential genome of *Pseudomonas aeruginosa* in cystic fibrosis sputum. Proc Natl Acad Sci U S A 112: 4110–4115. 10.1073/pnas.1419677112 25775563 PMC4386324

[8] Valentino MD, Foulston L, Sadaka A, Kos VN, Villet RA, et al (2014) Genes contributing to *Staphylococcus aureus* fitness in abscess- and infection-related ecologies. MBio 5: e01729–01714. 10.1128/mBio.01729-14 25182329 PMC4173792

[9] Lew DP, Waldvogel FA (2004) Osteomyelitis. Lancet 364: 369–379. 15276398 10.1016/S0140-6736(04)16727-5

[10] Rubin RJ, Harrington CA, Poon A, Dietrich K, Greene JA, et al (1999) The economic impact of *Staphylococcus aureus* infection in New York City hospitals. Emerg Infect Dis 5: 9–17. 10081667 10.3201/eid0501.990102 PMC2627695

[11] Spencer JA, Ferraro F, Roussakis E, Klein A, Wu J, et al (2014) Direct measurement of local oxygen concentration in the bone marrow of live animals. Nature 508: 269–273. 10.1038/nature13034 24590072 PMC3984353

[12] Harrison JS, Rameshwar P, Chang V, Bandari P (2002) Oxygen saturation in the bone marrow of healthy volunteers. Blood 99: 394 11783438 10.1182/blood.v99.1.394

[13] Lu C, Saless N, Wang X, Sinha A, Decker S, et al (2013) The role of oxygen during fracture healing. Bone 52: 220–229. 10.1016/j.bone.2012.09.037 23063782 PMC4827706

[14] Bar-Shavit Z (2008) Taking a toll on the bones: regulation of bone metabolism by innate immune regulators. Autoimmunity 41: 195–203. 10.1080/08916930701694469 18365832

[15] Wright JA, Nair SP (2010) Interaction of staphylococci with bone. Int J Med Microbiol 300: 193–204. 10.1016/j.ijmm.2009.10.003 19889575 PMC2814006

[16] Takayanagi H (2009) Osteoimmunology and the effects of the immune system on bone. Nat Rev Rheumatol 5: 667–676. 10.1038/nrrheum.2009.217 19884898

[17] Gerber JS, Coffin SE, Smathers SA, Zaoutis TE (2009) Trends in the incidence of methicillin-resistant *Staphylococcus aureus* infection in children's hospitals in the United States. Clin Infect Dis 49: 65–71. 10.1086/599348 19463065 PMC2897056

[18] Carrillo-Marquez MA, Hulten KG, Hammerman W, Mason EO, Kaplan SL (2009) USA300 is the predominant genotype causing *Staphylococcus aureus* septic arthritis in children. Pediatr Infect Dis J 28: 1076–1080. 10.1097/INF.0b013e3181adbcfe 19820424

[19] Subashchandrabose S, Smith SN, Spurbeck RR, Kole MM, Mobley HL (2013) Genome-wide detection of fitness genes in uropathogenic *Escherichia coli* during systemic infection. PLoS Pathog 9: e1003788 10.1371/journal.ppat.1003788 24339777 PMC3855560

[20] Kamp HD, Patimalla-Dipali B, Lazinski DW, Wallace-Gadsden F, Camilli A (2013) Gene fitness landscapes of *Vibrio cholerae* at important stages of its life cycle. PLoS Pathog 9: e1003800 10.1371/journal.ppat.1003800 24385900 PMC3873450

[21] Wong SM, Bernui M, Shen H, Akerley BJ (2013) Genome-wide fitness profiling reveals adaptations required by *Haemophilus* in coinfection with influenza A virus in the murine lung. Proc Natl Acad Sci U S A 110: 15413–15418. 10.1073/pnas.1311217110 24003154 PMC3780910

[22] Palace SG, Proulx MK, Lu S, Baker RE, Goguen JD (2014) Genome-wide mutant fitness profiling identifies nutritional requirements for optimal growth of *Yersinia pestis* in deep tissue. MBio 5. 10.1128/mBio.01385-14 PMC4147864 25139902

[23] Skurnik D, Roux D, Aschard H, Cattoir V, Yoder-Himes D, et al (2013) A comprehensive analysis of *in vitro* and *in vivo* genetic fitness of *Pseudomonas aeruginosa* using high-throughput sequencing of transposon libraries. PLoS Pathog 9: e1003582 10.1371/journal.ppat.1003582 24039572 PMC3764216

[24] Peschel A, Jack RW, Otto M, Collins LV, Staubitz P, et al (2001) *Staphylococcus aureus* resistance to human defensins and evasion of neutrophil killing via the novel virulence factor MprF is based on modification of membrane lipids with l-lysine. J Exp Med 193: 1067–1076. 11342591 10.1084/jem.193.9.1067 PMC2193429

[25] Mazmanian SK, Skaar EP, Gaspar AH, Humayun M, Gornicki P, et al (2003) Passage of heme-iron across the envelope of *Staphylococcus aureus* . Science 299: 906–909. 12574635 10.1126/science.1081147

[26] Kinkel TL, Roux CM, Dunman PM, Fang FC (2013) The *Staphylococcus aureus* SrrAB two-component system promotes resistance to nitrosative stress and hypoxia. MBio 4: e00696–00613. 10.1128/mBio.00696-13 24222487 PMC3892780

[27] Pragman AA, Yarwood JM, Tripp TJ, Schlievert PM (2004) Characterization of virulence factor regulation by SrrAB, a two-component system in *Staphylococcus aureus* . J Bacteriol 186: 2430–2438. 15060046 10.1128/JB.186.8.2430-2438.2004 PMC412142

[28] Carreau A, El Hafny-Rahbi B, Matejuk A, Grillon C, Kieda C (2011) Why is the partial oxygen pressure of human tissues a crucial parameter? Small molecules and hypoxia. J Cell Mol Med 15: 1239–1253. 10.1111/j.1582-4934.2011.01258.x 21251211 PMC4373326

[29] Throup JP, Zappacosta F, Lunsford RD, Annan RS, Carr SA, et al (2001) The srhSR gene pair from *Staphylococcus aureus*: genomic and proteomic approaches to the identification and characterization of gene function. Biochemistry 40: 10392–10401. 11513618 10.1021/bi0102959

[30] Vitko NP, Spahich NA, Richardson AR (2015) Glycolytic dependency of high-level nitric oxide resistance and virulence in *Staphylococcus aureus* . MBio 6. 10.1128/mBio.00045-15 PMC4453550 25852157

[31] Daley JM, Thomay AA, Connolly MD, Reichner JS, Albina JE (2008) Use of Ly6G-specific monoclonal antibody to deplete neutrophils in mice. J Leukoc Biol 83: 64–70. 17884993 10.1189/jlb.0407247

[32] Cassat JE, Hammer ND, Campbell JP, Benson MA, Perrien DS, et al (2013) A secreted bacterial protease tailors the *Staphylococcus aureus* virulence repertoire to modulate bone remodeling during osteomyelitis. Cell Host Microbe 13: 759–772. 10.1016/j.chom.2013.05.003 23768499 PMC3721972

[33] Hammer ND, Cassat JE, Noto MJ, Lojek LJ, Chadha AD, et al (2014) Inter- and intraspecies metabolite exchange promotes virulence of antibiotic-resistant *Staphylococcus aureus* . Cell Host Microbe 16: 531–537. 10.1016/j.chom.2014.09.002 25299336 PMC4197139

[34] Geisinger E, Muir TW, Novick RP (2009) agr receptor mutants reveal distinct modes of inhibition by staphylococcal autoinducing peptides. Proc Natl Acad Sci U S A 106: 1216–1221. 10.1073/pnas.0807760106 19147840 PMC2633565

[35] Queck SY, Jameson-Lee M, Villaruz AE, Bach TH, Khan BA, et al (2008) RNAIII-independent target gene control by the *agr* quorum-sensing system: insight into the evolution of virulence regulation in *Staphylococcus aureus* . Mol Cell 32: 150–158. 10.1016/j.molcel.2008.08.005 18851841 PMC2575650

[36] Berube BJ, Sampedro GR, Otto M, Bubeck Wardenburg J (2014) The *psmalpha* locus regulates production of *Staphylococcus aureus* alpha-toxin during infection. Infect Immun 82: 3350–3358. 10.1128/IAI.00089-14 24866799 PMC4136214

[37] Yarwood JM, Bartels DJ, Volper EM, Greenberg EP (2004) Quorum sensing in *Staphylococcus aureus* biofilms. J Bacteriol 186: 1838–1850. 14996815 10.1128/JB.186.6.1838-1850.2004 PMC355980

[38] Yarwood JM, McCormick JK, Schlievert PM (2001) Identification of a novel two-component regulatory system that acts in global regulation of virulence factors of *Staphylococcus aureus* . J Bacteriol 183: 1113–1123. 11157922 10.1128/JB.183.4.1113-1123.2001 PMC94983

[39] Rothfork JM, Timmins GS, Harris MN, Chen X, Lusis AJ, et al (2004) Inactivation of a bacterial virulence pheromone by phagocyte-derived oxidants: new role for the NADPH oxidase in host defense. Proc Natl Acad Sci U S A 101: 13867–13872. 15353593 10.1073/pnas.0402996101 PMC518845

[40] Sun F, Liang H, Kong X, Xie S, Cho H, et al (2012) Quorum-sensing *agr* mediates bacterial oxidation response via an intramolecular disulfide redox switch in the response regulator AgrA. Proc Natl Acad Sci U S A 109: 9095–9100. 10.1073/pnas.1200603109 22586129 PMC3384213

[41] Ingavale SS, Van Wamel W, Cheung AL (2003) Characterization of RAT, an autolysis regulator in *Staphylococcus aureus* . Mol Microbiol 48: 1451–1466. 12791130 10.1046/j.1365-2958.2003.03503.x

[42] Pagels M, Fuchs S, Pane-Farre J, Kohler C, Menschner L, et al (2010) Redox sensing by a Rex-family repressor is involved in the regulation of anaerobic gene expression in *Staphylococcus aureus* . Mol Microbiol 76: 1142–1161. 10.1111/j.1365-2958.2010.07105.x 20374494 PMC2883068

[43] Chen PR, Bae T, Williams WA, Duguid EM, Rice PA, et al (2006) An oxidation-sensing mechanism is used by the global regulator MgrA in *Staphylococcus aureus* . Nat Chem Biol 2: 591–595. 16980961 10.1038/nchembio820

[44] Fujimoto DF, Higginbotham RH, Sterba KM, Maleki SJ, Segall AM, et al (2009) *Staphylococcus aureus* SarA is a regulatory protein responsive to redox and pH that can support bacteriophage lambda integrase-mediated excision/recombination. Mol Microbiol 74: 1445–1458. 10.1111/j.1365-2958.2009.06942.x 19919677 PMC2879156

[45] Sun F, Ji Q, Jones MB, Deng X, Liang H, et al (2012) AirSR, a [2Fe-2S] cluster-containing two-component system, mediates global oxygen sensing and redox signaling in *Staphylococcus aureus* . J Am Chem Soc 134: 305–314. 10.1021/ja2071835 22122613 PMC3257388

[46] Boles BR, Thoendel M, Roth AJ, Horswill AR (2010) Identification of genes involved in polysaccharide-independent *Staphylococcus aureus* biofilm formation. PLoS One 5: e10146 10.1371/journal.pone.0010146 20418950 PMC2854687

[47] Kaito C, Saito Y, Nagano G, Ikuo M, Omae Y, et al (2011) Transcription and translation products of the cytolysin gene *psm-mec* on the mobile genetic element SCCmec regulate *Staphylococcus aureus* virulence. PLoS Pathog 7: e1001267 10.1371/journal.ppat.1001267 21304931 PMC3033363

[48] Fey PD, Endres JL, Yajjala VK, Widhelm TJ, Boissy RJ, et al (2013) A genetic resource for rapid and comprehensive phenotype screening of nonessential *Staphylococcus aureus* genes. MBio 4: e00537–00512. 23404398 10.1128/mBio.00537-12 PMC3573662

[49] Yanisch-Perron C, Vieira J, Messing J (1985) Improved M13 phage cloning vectors and host strains: nucleotide sequences of the M13mp18 and pUC19 vectors. Gene 33: 103–119. 2985470 10.1016/0378-1119(85)90120-9

[50] Chen J, Novick RP (2007) *svrA*, a multi-drug exporter, does not control *agr* . Microbiology 153: 1604–1608. 17464075 10.1099/mic.0.2007/006247-0

[51] Bubeck Wardenburg J, Williams WA, Missiakas D (2006) Host defenses against *Staphylococcus aureus* infection require recognition of bacterial lipoproteins. Proc Natl Acad Sci U S A 103: 13831–13836. 16954184 10.1073/pnas.0603072103 PMC1564215

[52] Mesak LR, Yim G, Davies J (2009) Improved *lux* reporters for use in *Staphylococcus aureus* . Plasmid 61: 182–187. 10.1016/j.plasmid.2009.01.003 19399993

[53] Dunman PM, Murphy E, Haney S, Palacios D, Tucker-Kellogg G, et al (2001) Transcription profiling-based identification of *Staphylococcus aureus* genes regulated by the *agr* and/or *sarA* loci. J Bacteriol 183: 7341–7353. 11717293 10.1128/JB.183.24.7341-7353.2001 PMC95583

[54] Beenken KE, Dunman PM, McAleese F, Macapagal D, Murphy E, et al (2004) Global gene expression in *Staphylococcus aureus* biofilms. J Bacteriol 186: 4665–4684. 15231800 10.1128/JB.186.14.4665-4684.2004 PMC438561

[55] Bischoff M, Dunman P, Kormanec J, Macapagal D, Murphy E, et al (2004) Microarray-based analysis of the *Staphylococcus aureus sigmaB* regulon. J Bacteriol 186: 4085–4099. 15205410 10.1128/JB.186.13.4085-4099.2004 PMC421609

[56] Anderson KL, Roberts C, Disz T, Vonstein V, Hwang K, et al (2006) Characterization of the *Staphylococcus aureus* heat shock, cold shock, stringent, and SOS responses and their effects on log-phase mRNA turnover. J Bacteriol 188: 6739–6756. 16980476 10.1128/JB.00609-06 PMC1595530

[57] Roberts C, Anderson KL, Murphy E, Projan SJ, Mounts W, et al (2006) Characterizing the effect of the *Staphylococcus aureus* virulence factor regulator, SarA, on log-phase mRNA half-lives. J Bacteriol 188: 2593–2603. 16547047 10.1128/JB.188.7.2593-2603.2006 PMC1428411

[58] Khodaverdian V, Pesho M, Truitt B, Bollinger L, Patel P, et al (2013) Discovery of antivirulence agents against methicillin-resistant *Staphylococcus aureus* . Antimicrob Agents Chemother 57: 3645–3652. 10.1128/AAC.00269-13 23689713 PMC3719762

