# Supplementary material for: Bacterial Hypoxic Responses Revealed as Critical Determinants of the Host-Pathogen Outcome by TnSeq Analysis of Staphylococcus aureus Invasive Infection
Source: PLoS Pathog. 2015 Dec 18;11(12):e1005341. doi: 10.1371/journal.ppat.1005341 (PMC4684308; doi:10.1371/journal.ppat.1005341)
Supplement: S1 Table — (PDF) [file ppat.1005341.s001.pdf]

8325 Locus	8325 Uniprot ID	8325 annotation	USA300 Locus	USA300 annotation	Product	Length	In vitro dval	osteo dval	Osteo stdev	dval_ratio	Compromised in vitro?	Essential in Abscess Infection[1]
SAOUHSC_00358	Q2G103	hypothetical protein	SAUSA300_0374	hypothetical protein	YP_498947.1	251	0.058	0.001	0.001	0.014	X	X
SAOUHSC_00460	Q2G1S0	hypothetical protein	N/A	N/A	YP_499039.1	155	0.102	0.001	0.001	0.010		
SAOUHSC_00473	Q2G0S1	hypothetical protein	N/A	N/A	YP_499052.1	110	0.048	0.007	0.009	0.135	X	
SAOUHSC_00541	Q2G0L9	tadA; tRNA-specific adenosine deaminase	SAUSA300_0543	tRNA-specific adenosine deaminase	YP_499113.1	470	0.011	0.000	0.000	0.000	X	X
SAOUHSC_00580	Q2G0I5	hypothetical protein	SAUSA300_0575	hypothetical protein	YP_499147.1	341	0.064	0.000	0.001	0.005	X	X
SAOUHSC_00650	Q2G2E3	hypothetical protein	N/A	N/A	YP_499209.1	92	0.133	0.001	0.001	0.005		
SAOUHSC_00680	Q2G0C5	hypothetical protein	N/A	N/A	YP_499239.1	95	0.571	0.001	0.002	0.002		
SAOUHSC_00841	Q2FZZ3	hypothetical protein	SAUSA300_0795	putative thioredoxin	YP_499395.1	296	0.044	0.000	0.000	0.000	X	X
SAOUHSC_00873	Q2FZW2	hypothetical protein	SAUSA300_0839	hypothetical protein	YP_499426.1	242	0.214	0.001	0.001	0.004		
SAOUHSC_00880	Q2FZV5	hypothetical protein	SAUSA300_0846	Na+/H+ antiporter family protein	YP_499433.1	1316	0.039	0.007	0.010	0.183	X	X
SAOUHSC_00919	Q2FZS1	hypothetical protein	SAUSA300_0884	hypothetical protein	YP_499472.1	185	0.069	0.000	0.001	0.004	X	
SAOUHSC_00964	Q2FZN6	hypothetical protein	SAUSA300_0931	hypothetical protein	YP_499517.1	176	0.015	0.010	0.018	0.701	X	X
SAOUHSC_01000	Q2FZK1	qoxC; cytochrome c oxidase subunit III	SAUSA300_0961	quinol oxidase, subunit III	YP_499552.1	605	0.029	0.008	0.008	0.269	X	
SAOUHSC_01034	Q2FZH1	hypothetical protein	SAUSA300_0988	trkA; potassium uptake protein	YP_499582.1	662	0.140	0.000	0.000	0.001		X
SAOUHSC_01087	Q2FZE4	iron compound ABC transporter permease	SAUSA300_1033	Probable heme-iron transport system permease protein IsdF	YP_499632.1	293	0.058	0.000	0.000	0.003	X	
SAOUHSC_01099	Q2FZD3	Endonuclease MutS2	SAUSA300_1043	MutS2 protein	YP_499643.1	2348	0.056	0.004	0.006	0.070	X	X
SAOUHSC_01193	Q2FZ58	hypothetical protein	SAUSA300_1119	hypothetical protein	YP_499732.1	1646	0.046	0.008	0.008	0.176	X	X
SAOUHSC_01206	Q2FZ47	UPF0122 protein	SAUSA300_1129	UPF0122 protein	YP_499743.1	332	0.016	0.010	0.017	0.605	X	X
SAOUHSC_01256	Q2FZ15	hypothetical protein	SAUSA300_1172	Peptidase, M16 family;	YP_499789.1	1286	0.024	0.010	0.009	0.417	X	
SAOUHSC_01289	Q2FYY4	hypothetical protein	N/A	N/A	YP_499820.1	224	0.010	0.000	0.000	0.000	X	
SAOUHSC_01304	Q2FYW9	hypothetical protein	SAUSA300_1211	hypothetical protein	YP_499835.1	194	0.030	0.000	0.000	0.000	X	
SAOUHSC_01325	Q2FYU9	hypothetical protein	N/A	N/A	YP_499855.1	104	0.010	0.000	0.000	0.000	X	X
SAOUHSC_01344	Q2FYT1	hypothetical protein	N/A	N/A	YP_499873.1	488	0.013	0.001	0.001	0.089	X	X
SAOUHSC_01359	Q2G2M2	mprF; Phosphatidylglycerol lysyltransferase	SAUSA300_1255	oxacillin resistance-related FmtC protein	YP_499886.1	2522	0.040	0.009	0.010	0.227	X	X
SAOUHSC_01367	Q2FYR8	anthranilate synthase component II	SAUSA300_1263	Anthranilate synthase, glutamine amidotransferase, component II; ; trpG	YP_499894.1	566	0.401	0.003	0.005	0.008		
SAOUHSC_01439	Q2FYK1	hypothetical protein	N/A	N/A	YP_499964.1	221	0.034	0.000	0.000	0.007	X	X
SAOUHSC_01500	Q2FYF2	hypothetical protein	N/A	N/A	YP_500018.1	116	0.483	0.001	0.002	0.002		
SAOUHSC_01549	Q2FYB5	transcriptional activator rinB-like protein	SAUSA300_1412	PhiSLT ORF 50-like protein	YP_500065.1	167	0.053	0.009	0.014	0.167	X	X
SAOUHSC_01555	Q2FYA9	Conserved hypothetical phage protein	N/A	N/A	YP_500071.1	347	0.070	0.000	0.000	0.002	X	
SAOUHSC_01585	Q2FY80	respiratory response protein SrrB	SAUSA300_1441	srrB; respiratory response protein	YP_500100.1	1751	1.195	0.010	0.005	0.008		X
SAOUHSC_01586	Q2FY79	Transcriptional regulatory protein SrrA	SAUSA300_1442	Staphylococcal respiratory response protein, SrrA	YP_500101.1	725	0.482	0.001	0.000	0.003		X
SAOUHSC_01595	Q2FY70	hypothetical protein	N/A	N/A	YP_500110.1	248	0.043	0.000	0.000	0.000	X	X
SAOUHSC_01621	Q2FY45	nusB; N utilization substance protein B homolog	SAUSA300_1473	N utilization substance protein B homolog; nusB	YP_500135.1	389	0.041	0.010	0.016	0.244	X	X
SAOUHSC_01645	Q2FY28	hypothetical protein	SAUSA300_1506	hypothetical protein	YP_500157.1	329	0.062	0.002	0.003	0.030	X	
SAOUHSC_01671	Q2FY03	Diacylglycerol kinase, putative	SAUSA300_1529	Diacylglycerol kinase; dgkA	YP_500182.1	344	0.172	0.004	0.008	0.026		X
SAOUHSC_01759	Q2FXS6	cell shape-determining protein MreC	SAUSA300_1605	Rod shape-determining protein MreC	YP_500264.1	842	0.021	0.005	0.008	0.233	X	
SAOUHSC_01770	Q2FXR5	hypothetical protein	N/A	N/A	YP_500275.1	101	0.065	0.000	0.000	0.000	X	
SAOUHSC_01804	Q2FXN1	Transposase, putative	N/A	N/A	YP_500309.1	806	0.065	0.009	0.014	0.140	X	
SAOUHSC_01812	Q2FXM3	hypothetical protein	SAUSA300_1650	hypothetical protein	YP_500317.1	941	0.116	0.000	0.000	0.004		
SAOUHSC_01853	Q2G246	hypothetical protein	N/A	N/A	YP_500357.1	104	0.658	0.002	0.001	0.003		
SAOUHSC_01902	Q2FXE7	hypothetical protein	N/A	N/A	YP_500403.1	302	0.026	0.001	0.001	0.028	X	X
SAOUHSC_01906	Q2FXE3	Truncated transposase, putative	N/A	N/A	YP_500407.1	608	0.021	0.003	0.005	0.137	X	
SAOUHSC_01930	Q2FXD3	hypothetical protein	SAUSA300_1749	hypothetical protein	YP_500431.1	359	0.080	0.001	0.000	0.011	X	X
SAOUHSC_01953	Q2FXB2	Gallidermin superfamily epiA, putative	SAUSA300_1767	Lantibiotic epidermin biosynthesis protein EpiA	YP_500452.1	143	0.635	0.004	0.001	0.006		
SAOUHSC_01967	Q2G2F1	ABC transporter, ATP-binding protein, putative	SAUSA300_1786	ABC transporter, ATP-binding protein EcsA	YP_500465.1	740	0.017	0.000	0.000	0.004	X	X
SAOUHSC_01977	Q2FXA0	UPF0342 protein	SAUSA300_1795	hypothetical protein	YP_500474.1	344	0.070	0.005	0.008	0.070	X	
SAOUHSC_01986	Q2FX91	hypothetical protein	SAUSA300_1803	hypothetical protein	YP_500483.1	152	0.129	0.001	0.001	0.006		X
SAOUHSC_02015	Q2FX81	hypothetical protein	N/A	N/A	YP_500512.1	101	0.217	0.000	0.000	0.000		
SAOUHSC_02053	Q2FX47	Transcriptional activator rinb-related protein	SAUSA300_1412	similar to transcriptional activator rinB	YP_500546.1	188	0.239	0.010	0.013	0.040		X
SAOUHSC_02059	Q2FX41	phi PVL orf 52-like protein	N/A	N/A	YP_500552.1	245	0.112	0.006	0.010	0.057		X
SAOUHSC_02126	Q2G2S0	purB; adenylosuccinate lyase	SAUSA300_1889	Adenylosuccinate lyase; purB	YP_500617.1	1295	0.948	0.003	0.001	0.003		X
SAOUHSC_02153	Q2FWW8	hypothetical protein	SAUSA300_1912	putative membrane protein	YP_500643.1	680	0.019	0.008	0.005	0.430	X	X
SAOUHSC_02222	Q2FWQ8	Conserved hypothetical phage protein	N/A	N/A	YP_500707.1	221	0.190	0.001	0.001	0.008		
SAOUHSC_02290	Q2FWJ8	hypothetical protein	N/A	N/A	YP_500772.1	155	0.032	0.000	0.000	0.000	X	
SAOUHSC_02333	Q2FWF8	Probable transglycosylase SceD	SAUSA300_2051	Probable transglycosylase sceD	YP_500812.1	695	0.095	0.000	0.000	0.002	X	
SAOUHSC_02340	Q2FWF1	atpC; ATP synthase epsilon chain	SAUSA300_2057	ATP synthase epsilon chain; atpC	YP_500819.1	404	0.029	0.000	0.000	0.005	X	X
SAOUHSC_02343	Q2FWE9	atpG; ATP synthase gamma chain	SAUSA300_2059	ATP synthase gamma chain; atpG	YP_500821.2	866	0.015	0.000	0.000	0.004	X	X
SAOUHSC_02346	Q2FWE7	atpH; ATP synthase subunit delta	SAUSA300_2061	ATP synthase subunit delta; atpH	YP_500823.1	539	0.041	0.000	0.001	0.008	X	X
SAOUHSC_02360	Q2FWD9	tdk; thymidine kinase	SAUSA300_2073	Thymidine kinase; tdk	YP_500836.1	599	0.027	0.002	0.003	0.069	X	X
SAOUHSC_02364	Q2FWD5	hypothetical protein	SAUSA300_2077	hypothetical protein	YP_500840.1	335	0.139	0.004	0.002	0.025		X
SAOUHSC_02378	Q2FWC0	hypothetical protein	N/A	N/A	YP_500855.1	128	0.068	0.000	0.001	0.006	X	
SAOUHSC_02602	Q2FVU0	hypothetical protein	N/A	N/A	YP_501063.1	359	0.575	0.002	0.001	0.003		
SAOUHSC_02707	Q2FVK4	hypothetical protein	N/A	N/A	YP_501169.1	182	0.159	0.000	0.001	0.002		X
SAOUHSC_02845	Q2FV89	hypothetical protein	SAUSA300_2474	hypothetical protein	YP_501303.1	344	0.040	0.000	0.000	0.004	X	X
SAOUHSC_A02811	Q2FUX6	hypothetical protein	N/A	N/A	YP_501420.1	167	0.130	0.001	0.001	0.005		

[1]See Valentino et al, PMID: 25182329
