# Supplementary material for: Bacterial Hypoxic Responses Revealed as Critical Determinants of the Host-Pathogen Outcome by TnSeq Analysis of Staphylococcus aureus Invasive Infection
Source: PLoS Pathog. 2015 Dec 18;11(12):e1005341. doi: 10.1371/journal.ppat.1005341 (PMC4684308; doi:10.1371/journal.ppat.1005341)
Supplement: S2 Table — (PDF) [file ppat.1005341.s002.pdf]

8325 Locus	8325 Uniprot ID	8325 annotation	USA300 locus	USA300 annotation	Accession number	Length	In vitro dval	Osteo avg_dval	Osteo stdev	dval ratio
SAOUHSC_00087	Q2G1M0	hypothetical protein	SAUSA300_RS00675	hypothetical protein	YP_498688.1	122	0.192	0.044	0.069	0.228
SAOUHSC_00179	Q2G1E6	hypothetical protein	SAUSA300_0212	Gfo/Idh/MocA family oxidoreductase	YP_498776.1	1076	0.264	0.085	0.031	0.324
SAOUHSC_00208	Q2G216	hypothetical protein	SAUSA300_RS01255	hypothetical protein	YP_498804.1	143	0.279	0.028	0.046	0.099
SAOUHSC_00331	Q2G1V7	hypothetical protein	SAUSA300_0350	Cro/CI family transcriptional regulator-like protein	YP_498920.1	203	0.382	0.043	0.073	0.113
SAOUHSC_00346	Q2G115	GTP-dependent nucleic acid-binding protein EngD	SAUSA300_0364	GTP-binding protein YchF	YP_498935.1	1097	0.139	0.028	0.025	0.200
SAOUHSC_00347	Q2G114	hypothetical protein	SAUSA300_0365	hypothetical protein	YP_498936.1	191	0.337	0.028	0.015	0.084
SAOUHSC_00350	Q2G111	30S ribosomal protein S18	SAUSA300_0368	rpsR; 30S ribosomal protein S18	YP_498939.1	242	0.459	0.042	0.033	0.092
SAOUHSC_00372	Q2G0Y9	xanthine phosphoribosyltransferase	SAUSA300_0386	xpt; xanthine phosphoribosyltransferase	YP_498961.1	578	0.507	0.047	0.076	0.093
SAOUHSC_00380	Q2G0Y2	hypothetical protein	N/A	N/A	YP_498968.1	260	0.109	0.077	0.133	0.706
SAOUHSC_00397	Q2G0X6	type I restriction-modification system, M subunit	SAUSA300_0405	hsdM; type I restriction-modification system, M subunit	YP_498984.1	1472	0.214	0.078	0.049	0.365
SAOUHSC_00414	Q2G0W0	hypothetical protein	SAUSA300_0427	hypothetical protein	YP_499000.1	362	0.100	0.047	0.050	0.476
SAOUHSC_00420	Q2G0V5	Transporter	SAUSA300_0432	hypothetical protein	YP_499005.1	1337	0.127	0.080	0.044	0.633
SAOUHSC_00463	Q2G0T1	rnmV; Ribonuclease M5	SAUSA300_0469	hypothetical protein	YP_499042.1	536	0.214	0.090	0.130	0.422
SAOUHSC_00464	Q2G0T0	rsmA; Ribosomal RNA small subunit methyltransferase A	SAUSA300_0470	ksgA; dimethyladenosine transferase	YP_499043.1	893	0.574	0.084	0.033	0.147
SAOUHSC_00483	Q2G0R3	S1 RNA binding domain protein	SAUSA300_0486	hypothetical protein	YP_499060.1	401	0.119	0.046	0.056	0.389
SAOUHSC_00488	Q2G0Q8	Cysteine synthase	SAUSA300_0491	cysK; cysteine synthase A	YP_499065.1	932	0.176	0.073	0.084	0.416
SAOUHSC_00656	Q2G0E9	hypothetical protein	SAUSA300_0637	dihydroxyacetone kinase subunit DhaL	YP_499215.1	584	0.528	0.077	0.057	0.145
SAOUHSC_00670	Q2G0D5	hypothetical protein	SAUSA300_0650	phosphate transporter family protein	YP_499229.1	1007	1.413	0.033	0.019	0.023
SAOUHSC_00685	Q2G0C0	hypothetical protein	SAUSA300_0663	hypothetical protein	YP_499244.1	395	0.378	0.090	0.058	0.238
SAOUHSC_00705	Q2G0A0	Cys-tRNA(Pro)/Cys-tRNA(Cys) deacylase	SAUSA300_0682	ybaK; ybaK/ebsC protein	YP_499264.1	482	0.302	0.034	0.031	0.112
SAOUHSC_00719	Q2G1X7	queE; 7-carboxy-7-deazaguanine synthase	SAUSA300_0695	radical activating enzyme family protein	YP_499278.1	713	0.149	0.058	0.082	0.389
SAOUHSC_00802	Q2G025	est; carboxylesterase	SAUSA300_0763	est	YP_499358.1	740	0.276	0.060	0.041	0.217
SAOUHSC_00809	Q2G018	hypothetical protein	N/A	N/A	YP_499365.1	155	0.892	0.093	0.094	0.104
SAOUHSC_00818	Q2G010	nuc; thermonuclease	SAUSA300_0776	nuc; thermonuclease	YP_499373.1	686	0.266	0.037	0.019	0.137
SAOUHSC_00826	Q2G1T2	hypothetical protein	SAUSA300_0781	hypothetical protein	YP_499381.1	236	0.199	0.013	0.022	0.064
SAOUHSC_00843	Q2FZZ1	hypothetical protein	SAUSA300_0797	ABC transporter permease	YP_499397.1	695	0.197	0.026	0.024	0.130
SAOUHSC_00951	Q2FZP9	Putative phosphoesterase	SAUSA300_0916	hypothetical protein	YP_499504.1	509	0.660	0.083	0.121	0.126
SAOUHSC_00979	Q2FZM1	hypothetical protein	SAUSA300_0943	acetyltransferase	YP_499532.1	551	0.135	0.089	0.091	0.663
SAOUHSC_01008	Q2FZJ5	purE; N5-carboxyaminoimidazole ribonucleotide mutase	SAUSA300_0966	purE; phosphoribosylaminoimidazole carboxylase, catalytic subunit	YP_499558.1	419	0.141	0.069	0.118	0.489
SAOUHSC_01030	Q2FZH4	hypothetical protein	SAUSA300_0985	hypothetical protein	YP_499579.1	233	0.318	0.022	0.036	0.069
SAOUHSC_01032	Q2FZH2	Cytochrome d ubiquinol oxidase, subunit II, putative	SAUSA300_0987	cytochrome D ubiquinol oxidase, subunit II; cydB	YP_499581.1	1019	0.314	0.078	0.051	0.248
SAOUHSC_01037	Q2FZG7	hypothetical protein	SAUSA300_RS05335	hypothetical protein	YP_499586.1	122	0.101	0.022	0.037	0.218
SAOUHSC_01040	Q2FZG4	Pyruvate dehydrogenase complex, E1 component, alpha subunit, putative	SAUSA300_0993	pdhA; pyruvate dehydrogenase E1 component, alpha subunit	YP_499589.1	1112	0.770	0.074	0.038	0.096
SAOUHSC_01044	Q2G2A2	UPF0223 protein	SAUSA300_0997	hypothetical protein	YP_499593.1	275	0.132	0.018	0.023	0.138
SAOUHSC_01049	Q2G2A8	Spermidine/putrescine ABC transporter, spermidine/putrescine-binding protein, putative	SAUSA300_1002	potD; spermidine/putrescine ABC transporter spermidine/putrescine-binding protein	YP_499598.1	1073	0.183	0.047	0.030	0.256
SAOUHSC_01054	Q2G2G7	UPF0637 protein	SAUSA300_1006	hypothetical protein	YP_499602.1	614	0.148	0.028	0.026	0.190
SAOUHSC_01064	Q2G2C1	pyruvate carboxylase	SAUSA300_1014	pyruvate carboxylase	YP_499610.1	3452	0.620	0.078	0.047	0.125
SAOUHSC_01098	Q2FZD4	DNA-dependent DNA polymerase beta chain, putative	SAUSA300_1042	hypothetical protein	YP_499642.1	1712	0.177	0.040	0.027	0.224
SAOUHSC_01110	Q2FZC2	fibrinogen-binding protein-like protein	SAUSA300_1052	fibrinogen-binding protein	YP_499654.1	329	0.170	0.097	0.165	0.573
SAOUHSC_01111	Q2FZC1	hypothetical protein	SAUSA300_RS05675	hypothetical protein	YP_499655.1	92	0.155	0.025	0.044	0.164
SAOUHSC_01122	Q2G1X4	hypothetical protein	SAUSA300_RS05725	membrane protein	YP_499666.1	146	0.276	0.036	0.059	0.129
SAOUHSC_01215	Q2FZ38	rnhB; ribonuclease HII	SAUSA300_1137	rnhB; ribonuclease HII	YP_499752.1	767	0.192	0.070	0.048	0.364
SAOUHSC_01216	Q2FZ37	sucC; succinyl-CoA synthetase subunit beta	SAUSA300_1138	Succinyl-CoA synthetase subunit beta; SucC	YP_499753.1	1166	0.102	0.035	0.043	0.346
SAOUHSC_01218	Q2FZ36	sucD; succinyl-CoA synthetase subunit alpha	SAUSA300_1139	Succinyl-CoA ligase [ADP-forming] subunit alpha; sucD	YP_499754.1	908	0.100	0.037	0.058	0.365
SAOUHSC_01284	Q2FYY8	hypothetical protein	SAUSA300_1199	hypothetical protein	YP_499816.1	1238	0.103	0.039	0.012	0.378
SAOUHSC_01292	Q2FYY1	hypothetical protein	SAUSA300_1204	hypothetical protein	YP_499823.1	185	0.487	0.064	0.047	0.132
SAOUHSC_01296	Q2FYX7	hypothetical protein	SAUSA300_1207	hypothetical protein	YP_499827.1	584	0.102	0.026	0.029	0.257
SAOUHSC_01302	Q2FYX1	hypothetical protein	SAUSA300_1209	hypothetical protein	YP_499833.1	134	0.249	0.021	0.023	0.084
SAOUHSC_01326	Q2FYU8	gamma-aminobutyrate permease	SAUSA300_1231	gamma-aminobutyrate permease	YP_499856.1	1454	1.059	0.076	0.037	0.071
SAOUHSC_01336	Q2FYT9	UPF0291 protein	SAUSA300_1238	DUF896 Superfamily, conserved in B. Subtilis (SOS response)	YP_499865.1	239	0.221	0.082	0.101	0.372
SAOUHSC_01343	Q2FYT2	hypothetical protein	SAUSA300_1243	sbcC; exonuclease	YP_499872.1	107	0.541	0.072	0.079	0.133
SAOUHSC_01356	Q2G2N4	glcT	SAUSA300_1253	glcT; transcription antiterminator	YP_499883.1	851	0.670	0.086	0.068	0.128
SAOUHSC_01369	Q2FYR6	trpC; indole-3-glycerol-phosphate synthase	SAUSA300_1265	trpC; indole-3-glycerol-phosphate synthase	YP_499896.1	782	0.178	0.015	0.012	0.082
SAOUHSC_01377	Q2FYQ8	Putative oligopeptide transport ATP-binding protein oppF2	SAUSA300_1273	opp-2F oligopeptide permease, ATP-binding protein	YP_499904.1	701	0.174	0.033	0.041	0.193
SAOUHSC_01388	Q2FYP7	hypothetical protein	N/A	N/A	YP_499915.1	107	0.505	0.022	0.038	0.044
SAOUHSC_01404	Q2FYN1	hypothetical protein	SAUSA300_RS07040	hypothetical protein	YP_499931.1	188	0.461	0.039	0.028	0.086
SAOUHSC_01413	Q2G2J8	hypothetical protein	SAUSA300_1302	ATPase family protein	YP_499940.1	791	0.271	0.070	0.035	0.258
SAOUHSC_01414	Q2G2J7	hypothetical protein	SAUSA300_1303	hypothetical protein	YP_499941.1	335	0.322	0.065	0.056	0.201
SAOUHSC_01416	Q2FYM2	odhB; Dihydrolipoyllysine-residue succinyltransferase component of 2-oxoglutarate dehydrogenase complex	SAUSA300_1305	sucB; dihydrolipoamide succinyltransferase	YP_499943.1	1268	0.166	0.097	0.086	0.587
SAOUHSC_01433	Q2FYK7	hypothetical protein	SAUSA300_1318	hypothetical protein	YP_499958.1	839	0.155	0.058	0.027	0.370
SAOUHSC_01443	Q2FYJ7	hypothetical protein	SAUSA300_1326	putative cell wall enzyme EbsB	YP_499968.1	401	0.394	0.072	0.013	0.182
SAOUHSC_01448	Q2FYJ5	Quinolone resistance protein NorB	SAUSA300_1328	putative drug transporter	YP_499970.1	1391	0.255	0.073	0.013	0.286
SAOUHSC_01459	Q2FYI7	hypothetical protein	SAUSA300_1335	hypothetical protein	YP_499978.1	332	0.183	0.021	0.034	0.114
SAOUHSC_01468	Q2FYH9	hypothetical protein	SAUSA300_1342	hypothetical protein	YP_499986.1	341	0.306	0.043	0.051	0.142
SAOUHSC_01471	Q2FYH6	asnS; Asparagine--tRNA ligase	SAUSA300_1345	asnC; asparaginyl-tRNA synthetase	YP_499989.1	1292	0.379	0.071	0.046	0.186
SAOUHSC_01484	Q2FYG8	hypothetical protein	SAUSA300_RS07400	membrane protein	YP_500002.1	200	0.215	0.014	0.023	0.065
SAOUHSC_01527	Q2G2K3	hypothetical protein	SAUSA300_1395	phiSLT ORF116b-like protein	YP_500044.1	350	0.296	0.090	0.084	0.304
SAOUHSC_01528	Q2G2K2	bacteriophage L54aIg-like domain-containing protein	SAUSA300_1396	phiSLT ORF151-like protein, major tail protein	YP_500045.1	455	0.199	0.050	0.023	0.250
SAOUHSC_01531	Q2G2S5	SLT orf 123-like protein	SAUSA300_1398	phiSLT ORF123-like protein	YP_500048.1	401	0.353	0.072	0.063	0.205
SAOUHSC_01539	Q2FYC5	terminase-small subunit	SAUSA300_1405	phiSLT ORF 101-like protein, terminase, small subunit	YP_500055.1	305	0.177	0.013	0.021	0.075
SAOUHSC_01558	Q2FYA6	PVL orf 51-like protein	SAUSA300_1953	phiPVL ORF051-like protein	YP_500074.1	242	0.246	0.096	0.107	0.393
SAOUHSC_01560	Q2FYA4	Conserved hypothetical phage protein	SAUSA300_1420	Conserved hypothetical phage protein	YP_500076.1	257	0.121	0.017	0.029	0.137
SAOUHSC_01567	Q2FY98	Conserved hypothetical phage protein	SAUSA300_1426	hypothetical protein	YP_500082.1	362	0.164	0.077	0.075	0.469
SAOUHSC_01584	Q2FY81	hypothetical protein	SAUSA300_1440	hypothetical protein	YP_500099.1	869	0.281	0.034	0.029	0.122
SAOUHSC_01603	Q2FY62	hypothetical protein	N/A	N/A	YP_500118.1	254	0.204	0.058	0.100	0.285
SAOUHSC_01610	Q2FY55	UPF0403 protein	SAUSA300_1463	hypothetical protein	YP_500125.1	437	0.220	0.077	0.096	0.351
SAOUHSC_01612	Q2FY53	2-oxoisovalerate dehydrogenase, E1 component, beta subunit, putative	SAUSA300_1465	2-oxoisovalerate dehydrogenase, E1 component, beta subunit	YP_500127.1	983	0.404	0.080	0.041	0.197
SAOUHSC_01655	Q2FY19	fur; ferric uptake regulation protein	SAUSA300_1514	fur; ferric uptake regulation protein	YP_500166.1	410	0.128	0.097	0.104	0.752
SAOUHSC_01673	Q2FY01	hypothetical protein	SAUSA300_1531	phoH; PhoH family phosphate starvation-induced protein	YP_500184.1	947	0.320	0.047	0.060	0.147
SAOUHSC_01676	Q2FXZ9	UPF0365 protein	SAUSA300_1533	hypothetical protein	YP_500186.1	989	0.171	0.082	0.055	0.479
SAOUHSC_01689	Q2FXY6	rpsT; 30S ribosomal protein S20	SAUSA300_1545	rpsT	YP_500199.1	251	0.123	0.011	0.015	0.090
SAOUHSC_01703	Q2FXX7	hypothetical protein	N/A	N/A	YP_500213.1	161	0.545	0.051	0.085	0.094
SAOUHSC_01723	Q2FXV8	recD2; ATP-dependent RecD-like DNA helicase	SAUSA300_1576	RecD/TraA family helicase	YP_500232.1	2477	0.121	0.079	0.058	0.649
SAOUHSC_01729	Q2FXV2	hypothetical protein	SAUSA300_1581	hypothetical protein	YP_500238.1	146	0.357	0.099	0.087	0.278
SAOUHSC_01732	Q2FXV0	hypothetical protein	SAUSA300_1583	hypothetical protein	YP_500240.1	350	0.147	0.013	0.018	0.091
SAOUHSC_01761	Q2FXS4	hypothetical protein	SAUSA300_1606	hypothetical protein	YP_500266.1	473	0.548	0.012	0.009	0.022
SAOUHSC_01768	Q2FXR7	hypothetical protein	SAUSA300_1612	tag; DNA-3-methyladenine glycosidase	YP_500273.1	560	0.438	0.092	0.100	0.210
SAOUHSC_01779	Q2FXQ6	tig; trigger factor	SAUSA300_1622	tig	YP_500284.1	1301	0.469	0.060	0.059	0.128
SAOUHSC_01810	Q2FXM5	NADP-dependent malic enzyme, putative	SAUSA300_1648	putative NADP-dependent malic enzyme	YP_500315.1	1229	0.140	0.089	0.105	0.638
SAOUHSC_01824	Q2FXL1	thiI; Probable tRNA sulfurtransferase	SAUSA300_1661	thiamine biosynthesis protein ThiI	YP_500329.1	1223	0.114	0.059	0.017	0.518
SAOUHSC_01834	Q2FXK0	hypothetical protein	SAUSA300_RS09120	hypothetical protein	YP_500340.1	95	0.123	0.023	0.040	0.187
SAOUHSC_01866	Q2FXI1	hypothetical protein	SAUSA300_1695	hypothetical protein	YP_500369.1	725	0.315	0.037	0.027	0.117
SAOUHSC_01868	Q2FXH9	Putative dipeptidase	SAUSA300_1697	dipeptidase PepV	YP_500371.1	1409	0.152	0.072	0.017	0.475
SAOUHSC_01869	Q2FXH8	hypothetical protein	SAUSA300_1698	hypothetical protein	YP_500372.1	422	0.206	0.014	0.022	0.066
SAOUHSC_01879	Q9RFJ6	HTH-type transcriptional regulator rot	SAUSA300_1708	accessory regulator Rot	YP_500382.1	401	0.131	0.065	0.020	0.498
SAOUHSC_01881	Q2FXG6	hypothetical protein	SAUSA300_0267	transposase	YP_500384.1	491	0.161	0.097	0.052	0.604
SAOUHSC_01935	Q2FXC8	serine protease SplF	SAUSA300_1753	serine protease SplF	YP_500436.1	719	0.176	0.058	0.038	0.332
SAOUHSC_01938	Q2FXC5	serine protease SplD	SAUSA300_1755	serine protease SplD	YP_500439.1	719	0.343	0.087	0.111	0.254
SAOUHSC_01969	Q2G2T0	hypothetical protein	SAUSA300_1788	hypothetical protein	YP_500467.1	365	0.277	0.052	0.024	0.186
SAOUHSC_01971	Q2G2S9	hypothetical protein	SAUSA300_1789	hypothetical protein	YP_500468.1	557	0.323	0.076	0.077	0.236
SAOUHSC_01972	Q2G2S6	Foldase protein PrsA	SAUSA300_1790	foldase protein PrsA	YP_500469.1	962	0.256	0.099	0.125	0.388
SAOUHSC_01974	Q2G2S8	hypothetical protein	SAUSA300_1792	hypothetical protein	YP_500471.1	2936	0.934	0.072	0.046	0.077
SAOUHSC_02007	Q2G2V8	hypothetical protein	SAUSA300_RS10110	hypothetical protein	YP_500504.1	230	0.610	0.083	0.142	0.136
SAOUHSC_02011	Q2G2G9	Regulatory protein RecX	SAUSA300_1854	recombination regulator RecX	YP_500508.1	818	0.212	0.045	0.062	0.215
SAOUHSC_02012	Q93Q23	Monofunctional glycosyltransferase	SAUSA300_1855	sgtB; glycosyltransferase	YP_500509.1	809	2.062	0.021	0.013	0.010
SAOUHSC_02013	P0A0K3	hypothetical protein	SAUSA300_1856	hypothetical protein	YP_500510.1	515	0.431	0.088	0.062	0.205
SAOUHSC_02020	Q2FX76	holin	N/A	N/A	YP_500517.1	437	0.177	0.093	0.023	0.526
SAOUHSC_02052	Q2FX48	Hypothetical phage protein	N/A	N/A	YP_500545.1	146	0.542	0.023	0.034	0.042
SAOUHSC_02076	Q2FX29	Phi PVL orf 38-like protein-related protein	SAUSA300_1429	phiSLT ORF53-like protein	YP_500569.1	161	0.208	0.091	0.071	0.440
SAOUHSC_02096	Q2FX11	hypothetical protein	SAUSA300_1863	hypothetical protein	YP_500587.1	275	0.185	0.012	0.010	0.062
SAOUHSC_02155	Q2FWW6	hypothetical protein	SAUSA300_1914	GntR family regulatory protein	YP_500645.1	380	0.201	0.078	0.106	0.389
SAOUHSC_02158	Q2FWW3	hypothetical protein	SAUSA300_1916	hypothetical protein	YP_500648.1	1286	0.508	0.049	0.033	0.096
SAOUHSC_02170	Q2FWV4	peptidoglycan hydrolase	SAUSA300_1921	truncated amidase	YP_500657.1	296	0.957	0.079	0.122	0.082
SAOUHSC_02174	Q2FWV1	Holin, phage phi LC3 family	SAUSA300_1924	Holin	YP_500660.1	254	0.296	0.046	0.042	0.155
SAOUHSC_02188	Q2FWT7	phage head-tail adaptor	SAUSA300_1936	hypothetical protein	YP_500674.1	335	0.366	0.098	0.096	0.268
SAOUHSC_02207	Q2FWS3	Phi PVL/orf 52-like protein	SAUSA300_1418	phiSLT ORF 82-like protein	YP_500692.1	254	0.254	0.013	0.019	0.050
SAOUHSC_02220	Q2FWR0	Phi ETA orf 18-like protein	N/A	N/A	YP_500705.1	551	0.245	0.076	0.044	0.311
SAOUHSC_02221	Q2FWQ9	Conserved hypothetical phage protein	N/A	N/A	YP_500706.1	779	0.130	0.044	0.054	0.340
SAOUHSC_02227	Q2FWQ3	Conserved hypothetical phage protein	N/A	N/A	YP_500712.1	140	0.278	0.021	0.035	0.074
SAOUHSC_02228	Q2FWQ2	Conserved hypothetical phage protein	N/A	N/A	YP_500713.1	197	0.328	0.039	0.065	0.119
SAOUHSC_02233	Q2FWP8	phi PVL orf 32-like protein	N/A	N/A	YP_500717.1	260	0.273	0.022	0.016	0.082
SAOUHSC_02268	Q2FWM1	Sucrose-6-phosphate dehydrogenase, putative	SAUSA300_1994	scrB; sucrose-6-phosphate hydrolase	YP_500749.1	1484	0.265	0.088	0.052	0.333
SAOUHSC_02275	Q2FWL4	hypothetical protein	N/A	N/A	YP_500756.1	128	0.114	0.023	0.040	0.203
SAOUHSC_02288	Q2FWK0	leuD; 3-isopropylmalate dehydratase small subunit	SAUSA300_2013	leuD; isopropylmalate isomerase small subunit	YP_500770.1	572	0.236	0.085	0.078	0.358
SAOUHSC_02352	Q2G2F5	UDP-N-acetylglucosamine 2-epimerase	SAUSA300_2065	UDP-GlcNAc 2-epimerase	YP_500828.1	1127	0.329	0.063	0.097	0.192
SAOUHSC_02353	Q2FWE6	upp; uracil phosphoribosyltransferase	SAUSA300_2066	upp	YP_500829.1	629	0.457	0.050	0.037	0.110
SAOUHSC_02354	Q2FWE5	glyA; serine hydroxymethyltransferase	SAUSA300_2067	glyA; serine hydroxymethyltransferase	YP_500830.1	1238	0.373	0.048	0.047	0.129
SAOUHSC_02355	Q2FWE4	UPF0340 protein	SAUSA300_2068	hypothetical protein	YP_500831.1	524	0.446	0.082	0.070	0.185
SAOUHSC_02358	Q2FWE1	prmC; Release factor glutamine methyltransferase	SAUSA300_2071	HemK family modification methylase	YP_500834.1	752	0.791	0.026	0.028	0.033
SAOUHSC_02394	Q2FWA4	hypothetical protein	SAUSA300_RS11585	hypothetical protein	YP_500871.1	92	0.336	0.092	0.160	0.275
SAOUHSC_02462	Q2FW55	hypothetical protein	N/A	N/A	YP_500930.1	167	0.122	0.014	0.024	0.112
SAOUHSC_02473	Q2FW44	hypothetical protein	N/A	N/A	YP_500941.1	92	0.571	0.037	0.063	0.065
SAOUHSC_02521	Q2FVZ9	hypothetical protein	SAUSA300_RS12190	hypothetical protein	YP_500986.1	131	0.178	0.019	0.032	0.107
SAOUHSC_02540	Q2FVY2	Molybdopterin converting factor moa, putative	SAUSA300_2222	molybdopterin converting factor, subunit 2	YP_501003.1	446	0.254	0.029	0.027	0.115
SAOUHSC_02543	Q2FVX9	moaC; Cyclic pyranopterin monophosphate synthase accessory protein	SAUSA300_2225	molybdenum cofactor biosynthesis protein MoaC	YP_501006.1	494	0.106	0.077	0.125	0.723
SAOUHSC_02559	Q2G2K6	ureB; urease subunit beta	SAUSA300_2239	ureB; urease subunit beta	YP_501021.1	410	0.150	0.055	0.020	0.364
SAOUHSC_02703	Q2FVK8	gpmA; 2,3-bisphosphoglycerate-dependent phosphoglycerate mutase	SAUSA300_2362	gpmA; phosphoglyceromutase	YP_501165.1	686	0.528	0.098	0.060	0.186
SAOUHSC_02805	Q2G207	hypothetical protein	N/A	N/A	YP_501264.1	302	0.270	0.040	0.045	0.149
SAOUHSC_02824	Q2FVA9	hypothetical protein	SAUSA300_2457	phospholipase/carboxylesterase family protein	YP_501283.1	593	0.105	0.080	0.036	0.765
SAOUHSC_02838	Q2FV96	hypothetical protein	SAUSA300_RS13695	hypothetical protein	YP_501296.1	98	0.346	0.029	0.050	0.084
SAOUHSC_02861	Q2FV75	Methylated-DNA--protein-cysteine methyltransferase, putative	SAUSA300_2485	methylated DNA-protein cysteine methyltransferase	YP_501317.1	521	0.224	0.036	0.029	0.159
SAOUHSC_A00332	Q2G125	hypothetical protein	SAUSA300_RS01880	hypothetical protein	YP_498925.1	158	0.292	0.072	0.063	0.247
SAOUHSC_A01079	Q2FZB5	hypothetical protein	SAUSA300_RS05700	hypothetical protein	YP_499661.1	155	1.143	0.072	0.112	0.063
SAOUHSC_A02013	Q2FX04	hypothetical protein	N/A	N/A	YP_500594.1	188	0.269	0.043	0.043	0.160
SAOUHSC_A02169	Q2FWK8	hypothetical protein	SAUSA300_RS11030	hypothetical protein	YP_500762.1	155	0.285	0.028	0.023	0.097
SAOUHSC_A02331	Q2FW60	hypothetical protein	SAUSA300_RS11885	hypothetical protein	YP_500925.1	161	0.367	0.051	0.084	0.138
SAOUHSC_A02503	Q2G2I4	hypothetical protein	SAUSA300_RS12725	hypothetical protein	YP_501097.1	269	0.610	0.092	0.157	0.150
SAOUHSC_T00046	tRNA-OTHER	tRNA				74	1.113	0.037	0.032	0.033
