# Supplementary material for: Bacterial Hypoxic Responses Revealed as Critical Determinants of the Host-Pathogen Outcome by TnSeq Analysis of Staphylococcus aureus Invasive Infection
Source: PLoS Pathog. 2015 Dec 18;11(12):e1005341. doi: 10.1371/journal.ppat.1005341 (PMC4684308; doi:10.1371/journal.ppat.1005341)
Supplement: S3 Table — (PDF) [file ppat.1005341.s003.pdf]

COL Locus	Annotation[1]	Fold Increase[2]	p-value	Regulated under nitrosative stress[3]
SA1124	ctaA; cytochrome oxidase assembly protein (ctaA) {Staphylococcus aureus COL}	4.2	0.00	
SA0965	ctaB;conserved cytochrome caa3 oxidase (assembly factor) homolog {N315}	2.0	0.00	
SA1094	cydA;cytochrome d ubiquinol oxidase, subunit I (cydA) {Staphylococcus aureus COL}	2.1	0.01	X
SA1622	glyS; glycyl-tRNA synthetase (glyS) {Staphylococcus aureus COL}	2.3	0.02	
SA1362	hom; homoserine dehydrogenase (hom) {Staphylococcus aureus COL}	3.1	0.02	
SA2635	nrdD; anaerobic ribonucleoside-triphosphate reductase (nrdD) {Staphylococcus aureus COL}	5.5	0.01	X
SA2634	nrdG; anaerobic ribonucleoside-triphosphate reductase activating protein (nrdG) {Staphylococcus aureus COL}	6.2	0.02	X
SA0205	pflA; pyruvate formate-lyase-activating enzyme (pflA) {Staphylococcus aureus COL}	4.2	0.04	X
SA0204	pflB; formate acetyltransferase (pflB) {Staphylococcus aureus COL}	3.8	0.02	X
SA0244	scdA; ScdA protein (scdA) {Staphylococcus aureus COL}	9.7	0.00	X
SA1363	thrC; threonine synthase (thrC) {Staphylococcus aureus COL}	3.1	0.03	
SA0218	conserved hypothetical protein {Staphylococcus aureus COL}	3.0	0.01	X
SA0219	hypothetical protein {Staphylococcus aureus COL}	2.8	0.00	X
SA0220	flavohemoprotein, putative {Staphylococcus aureus COL}	2.6	0.00	X
SA0872	OsmC/Ohr family protein {Staphylococcus aureus COL}	2.5	0.01	
SA0910	conserved hypothetical protein, similar to quinol oxidase polypeptide IV QoxD	2.4	0.01	
SA0959	NADH-dependent flavin oxidoreductase, Oye family {Staphylococcus aureus COL}	3.1	0.02	
SA1126	conserved hypothetical protein {Staphylococcus aureus COL}	2.1	0.01	X
SA1360	aspartate kinase {Staphylococcus aureus COL}	6.8	0.01	
SA1705	hypothetical protein {Staphylococcus aureus COL}	2.0	0.01	
SA2192	conserved hypothetical protein {MRSA252, Mu50, MW2, MSSA476, N315}	2.4	0.02	
SA2563	ATP-dependent Clp protease, putative {Staphylococcus aureus COL}	3.4	0.02	X
SA2571	conserved hypothetical protein {Staphylococcus aureus COL}	2.9	0.00	
SA2626	conserved hypothetical protein {Staphylococcus aureus COL}	2.3	0.04	
SAV1941	putative membrane protein {MRSA252, MSSA476, MW2, Mu50}	2.1	0.01	
SA2338	hypothetical protein {Staphylococcus aureus COL}	-2.1	0.04	
SA1758	Ald; alanine dehydrogenase (ald) {Staphylococcus aureus COL}	-2.1	0.01	
SA2198	aldC; alpha-acetolactate decarboxylase (aldC) {Staphylococcus aureus COL}	-2.6	0.03	
SA1062	atl; bifunctional autolysin (atl) {Staphylococcus aureus COL}	-2.1	0.03	
SA2199	budB; acetolactate synthase, catabolic (budB) {Staphylococcus aureus COL}	-2.5	0.02	
SA0138	cap5C; capsular polysaccharide biosynthesis protein Cap5C (cap5C) {Staphylococcus aureus COL}	-2.1	0.00	
SA1595	gcvT; glycine cleavage system T protein (gcvT) {Staphylococcus aureus COL}	-3.6	0.00	
SA1742	gltA; citrate synthase (gltA) {Staphylococcus aureus COL}	-2.3	0.01	
SA0008	hutH; histidine ammonia-lyase {N315}	-2.3	0.04	
SA2323	hutI; imidazolonepropionase (hutI) {Staphylococcus aureus COL}	-3.4	0.04	
SA2324	hutU; urocanate hydratase (hutU) {Staphylococcus aureus COL}	-3.8	0.03	
SA2462	icaC; intercellular adhesion protein C {MRSA252, MSSA476, MW2, N315}	-2.7	0.01	
SA1741	icd; isocitrate dehydrogenase, NADP-dependent (icd) {Staphylococcus aureus COL}	-2.0	0.00	
SA0312	nanA; N-acetylneuraminate lyase (nanA) {Staphylococcus aureus COL}	-2.3	0.00	
SA0585	rplJ; ribosomal protein L10 (rplJ) {Staphylococcus aureus COL}	-2.3	0.04	
SA0211	acetyl-CoA acetyltransferase {Staphylococcus aureus COL}	-6.7	0.01	
SA0212	3-hydroxyacyl-CoA dehydrogenase protein {Staphylococcus aureus COL}	-8.6	0.03	
SA0213	acyl-CoA dehydrogenase family protein {Staphylococcus aureus COL}	-8.0	0.02	
SA0214	long-chain-fatty-acid--CoA ligase, putative {Staphylococcus aureus COL}	-8.0	0.02	
SA0215	propionate CoA-transferase, putative {Staphylococcus aureus COL}	-4.8	0.02	
SA0265	hypothetical protein {Staphylococcus aureus COL}	-2.1	0.02	
SA0267	hypothetical protein {Staphylococcus aureus COL}	-2.8	0.04	
SA0278	hypothetical protein {Staphylococcus aureus COL}	-2.3	0.04	
SA0299	hypothetical protein {Staphylococcus aureus COL}	-2.3	0.03	
SA0300	hypothetical protein {Staphylococcus aureus COL}	-2.2	0.01	
SA0311	sodium:solute symporter family protein {Staphylococcus aureus COL}	-2.5	0.04	
SA0599	conserved hypothetical protein {Staphylococcus aureus COL}	-2.6	0.01	
SA1225	hypothetical protein {Staphylococcus aureus COL}	-2.5	0.05	
SA1593	glycine cleavage system P protein, subunit 2 {Staphylococcus aureus COL}	-3.4	0.00	
SA1594	glycine cleavage system P protein, subunit 1 {Staphylococcus aureus COL}	-3.3	0.00	
SA1659	conserved hypothetical protein {Staphylococcus aureus COL}	-2.4	0.05	
SA1660	LamB/YcsF family protein {Staphylococcus aureus COL}	-2.0	0.03	
SA1661	acetyl-CoA carboxylase, biotin carboxylase, putative {Staphylococcus aureus COL}	-2.0	0.01	
SA1662	acetyl-CoA carboxylase, biotin carboxyl carrier protein, putative {Staphylococcus aureus COL}	-2.2	0.03	
SA1663	urea amidolyase-related protein {Staphylococcus aureus COL}	-2.2	0.04	
SA1996	ABC transporter, ATP-binding protein {Staphylococcus aureus COL}	-2.6	0.04	
SA1997	transcriptional regulator, GntR family {Staphylococcus aureus COL}	-2.3	0.03	
SA2521	transporter, putative {Staphylococcus aureus COL}	-2.0	0.02	
SA2636	citrate transporter, permease protein {Staphylococcus aureus COL}	-2.3	0.01	

[1] Annotation obtain from the COL genome unless otherwise noted.

[2] Fold increase is the ratio of transcript abundance in WT relative to the srrA mutant; Grey shading indicates that fold increase is estimated because the transcript was below threshold in the comparator c

[3] See Kinkel et al, PMID: 24222487

[4] "C" denotes compromised during TnSeq analysis of osteomyelitis
