# Supplementary material for: Bacterial Hypoxic Responses Revealed as Critical Determinants of the Host-Pathogen Outcome by TnSeq Analysis of Staphylococcus aureus Invasive Infection
Source: PLoS Pathog. 2015 Dec 18;11(12):e1005341. doi: 10.1371/journal.ppat.1005341 (PMC4684308; doi:10.1371/journal.ppat.1005341)
Supplement: S4 Table — (PDF) [file ppat.1005341.s004.pdf]

COL Locus	Annotation[1]	Fold Increase[2]	p-value	Regulated under nitrosative stress[3]	TnSeq Analysis[4]
SA0910	conserved hypothetical protein, similar to quinol oxidase polypeptide IV QoxD	3.8	0.01		
SA1478	Ald; alanine dehydrogenase (ald) {Staphylococcus aureus COL}	4.7	0.00		
SA1181	arcB; ornithine carbamoyltransferase (arcB) {Staphylococcus aureus COL}	21.5	0.02		
SA1182	arc; carbamate kinase (arcC) {Staphylococcus aureus COL}	12.1	0.03		
SA1124	ctaA; cytochrome oxidase assembly protein (ctaA) {Staphylococcus aureus COL}	7.3	0.03		
SA0965	ctaB; conserved cytochrome caa3 oxidase (assembly factor) homolog {N315}	4.2	0.01		
SA1719	hemA; glutamyl-tRNA reductase (hemA) {Staphylococcus aureus COL}	2.1	0.03	X	
SA1362	hom; homoserine dehydrogenase (hom) {Staphylococcus aureus COL}	3.9	0.02		
SA2618	idh; L-lactate dehydrogenase (ldh) {Staphylococcus aureus COL}	5.0	0.02		
SA1477	ilvA; threonine dehydratase, catabolic (ilvA) {Staphylococcus aureus COL}	3.5	0.01		
SA0600	ilvE; branched-chain amino acid aminotransferase (ilvE) {Staphylococcus aureus C	2.1	0.04		
SA0245	lytS; sensor histidine kinase LytS (lytS) {Staphylococcus aureus COL}	2.4	0.03		
SA2635	nrdD; anaerobic ribonucleoside-triphosphate reductase (nrdD) {Staphylococcus aur	35.9	0.00	X	
SA2634	nrdG; anaerobic ribonucleoside-triphosphate reductase activating protein (nrdG) {S	53.6	0.00	X	
SA0860	nuc; thermonuclease precursor (nuc) {Staphylococcus aureus COL}	2.1	0.04		C
SA0205	pflA; pyruvate formate-lyase-activating enzyme (pflA) {Staphylococcus aureus COL	8.2	0.00	X	
SA0204	pflB; formate acetyltransferase (pflB) {Staphylococcus aureus COL}	7.4	0.02	X	
SA1977	pheA; prephenate dehydratase (pheA) {Staphylococcus aureus COL}	6.2	0.01		
SA0078	plc; 1-phosphatidylinositol phosphodiesterase (plc) {Staphylococcus aureus COL}	9.9	0.00		
SA0244	scdA; ScdA protein (scdA) {Staphylococcus aureus COL}	37.4	0.00	X	
SA1868	splB; serine protease SplB (splB) {Staphylococcus aureus COL}	3.0	0.03		
SA1364	thrB; homoserine kinase (thrB) {Staphylococcus aureus COL}	5.5	0.03		
SA0089	antigen, 67 kDa {Staphylococcus aureus COL}	11.3	0.01	X	
SA0135	alcohol dehydrogenase, iron-containing {Staphylococcus aureus COL}	2.9	0.00		
SA0176	conserved hypothetical protein {Staphylococcus aureus COL}	3.4	0.01		
SA0177	glucokinase regulator-related protein {Staphylococcus aureus COL}	3.5	0.02		
SA0178	PTS system, IIBC components {Staphylococcus aureus COL}	3.0	0.02		
SA0202	sensor histidine kinase family protein {Staphylococcus aureus COL}	3.3	0.03		
SA0203	iron compound ABC transporter, iron compound-binding protein, putative {Staphyloc	3.6	0.02		
SA0207	conserved domain protein {Staphylococcus aureus COL}	3.3	0.00		
SA0217	ABC transporter, substrate-binding protein {Staphylococcus aureus COL}	7.5	0.02	X	
SA0218	conserved hypothetical protein {Staphylococcus aureus COL}	8.5	0.00	X	
SA0219	hypothetical protein {Staphylococcus aureus COL}	5.2	0.04	X	
SA0220	flavohemoprotein, putative {Staphylococcus aureus COL}	3.9	0.03	X	
SA0517	alpha-amylase family protein {Staphylococcus aureus COL}	6.3	0.02		
SA0518	transcriptional regulator, GntR family {Staphylococcus aureus COL}	6.3	0.01		
SA0602	hydrolase, haloacid dehalogenase-like family {Staphylococcus aureus COL}	2.8	0.05		
SA0660	alcohol dehydrogenase, zinc-containing {Staphylococcus aureus COL}	3.9	0.01	X	
SA0748	oxidoreductase, aldo/keto reductase family {Staphylococcus aureus COL}	2.1	0.03		
SA0871	acetyltransferase, putative {Staphylococcus aureus COL}	2.9	0.00		
SA0962	glycerophosphoryl diester phosphodiesterase GlpQ, putative {Staphylococcus aure	5.0	0.05		
SA1126	conserved hypothetical protein {Staphylococcus aureus COL}	4.1	0.01	X	
SA1308	pyruvate ferredoxin oxidoreductase, alpha subunit {Staphylococcus aureus COL}	2.4	0.04		
SA1358	conserved hypothetical protein {Staphylococcus aureus COL}	2.0	0.03		
SA1475	drug transporter, putative {Staphylococcus aureus COL}	4.1	0.02		C
SA1476	amino acid permease {Staphylococcus aureus COL}	5.8	0.01		
SA1866	serine protease SplD, putative {Staphylococcus aureus COL}	2.4	0.03		C
SA1976	nitric-oxide synthase, oxygenase subunit {Staphylococcus aureus COL}	6.8	0.01		
SA2192	conserved hypothetical protein {MRSA252, Mu50, MW2, MSSA476, N315}	11.9	0.03		
SA2401	formate/nitrite transporter family protein {Staphylococcus aureus COL}	3.1	0.02		
SA2496	conserved hypothetical protein {N315}	7.9	0.02		
SA2563	ATP-dependent Clp protease, putative {Staphylococcus aureus COL}	7.2	0.00	X	
SA2626	conserved hypothetical protein {Staphylococcus aureus COL}	2.3	0.03		
SA2662	transcriptional antiterminator, BglG family {Staphylococcus aureus COL}	4.8	0.02		
SA2732	transcriptional regulator, putative {Staphylococcus aureus COL}	6.3	0.01		
SA2734	conserved hypothetical protein {Staphylococcus aureus COL}	9.8	0.00		
SA2554.1	cidA; LrgA family protein {Staphylococcus aureus COL}	-7.7	0.04	X	
SA1742	gltA; citrate synthase (gltA) {Staphylococcus aureus COL}	-2.3	0.05		
SA0162	conserved NAD-dependent formate dehydrogenase {all strains, COL}	-3.7	0.04		
SA0211	acetyl-CoA acetyltransferase {Staphylococcus aureus COL}	-4.1	0.01		
SA0212	3-hydroxyacyl-CoA dehydrogenase protein {Staphylococcus aureus COL}	-3.8	0.05		
SA0213	acyl-CoA dehydrogenase family protein {Staphylococcus aureus COL}	-5.6	0.01		
SA0214	long-chain-fatty-acid--CoA ligase, putative {Staphylococcus aureus COL}	-7.9	0.02		
SA0215	propionate CoA-transferase, putative {Staphylococcus aureus COL}	-7.1	0.01		
SA0277	hypothetical protein {Staphylococcus aureus COL}	-2.3	0.02		
SA0486	staphylococcus tandem lipoprotein {Staphylococcus aureus COL}	-2.1	0.03		
SA0599	conserved hypothetical protein {Staphylococcus aureus COL}	-2.5	0.01		
SA0648	conserved hypothetical protein {N315}	-2.1	0.04		
SA1093	conserved hypothetical protein {Staphylococcus aureus COL}	-2.8	0.00		C
SA1189	acetyltransferase (GNAT) family protein {COL}	-2.4	0.01		
SA1350	conserved hypothetical protein {Staphylococcus aureus COL}	-2.8	0.01		
SA1532	hypothetical protein {Staphylococcus aureus COL}	-2.6	0.01		
SA1533	hypothetical protein {Staphylococcus aureus COL}	-2.5	0.02		C
SA2338	hypothetical protein {Staphylococcus aureus COL}	-2.2	0.01		
SA2348	drug transporter, putative {Staphylococcus aureus COL}	-2.6	0.04		
SA2521	transporter, putative {Staphylococcus aureus COL}	-2.9	0.00		
SA2609	conserved hypothetical protein {Staphylococcus aureus COL}	-2.2	0.02		
SA2717	hypothetical protein {Staphylococcus aureus COL}	-3.0	0.01		

[1] Annotation obtain from the COL genome unless otherwise noted.

[2] Fold increase is the ratio of transcript abundance in WT relative to the srrA mutant; Grey shading indicates that fold increase is estimated because the transcript was below threshold in the comparator condition.

[3] See Kinkel et al, PMID: 24222487

[4] "C" denotes compromised during TnSeq analysis of osteomyelitis
