# Supplementary figures and images for: Bacterial Hypoxic Responses Revealed as Critical Determinants of the Host-Pathogen Outcome by TnSeq Analysis of Staphylococcus aureus Invasive Infection
Source: PLoS Pathog. 2015 Dec 18;11(12):e1005341. doi: 10.1371/journal.ppat.1005341 (PMC4684308; doi:10.1371/journal.ppat.1005341)

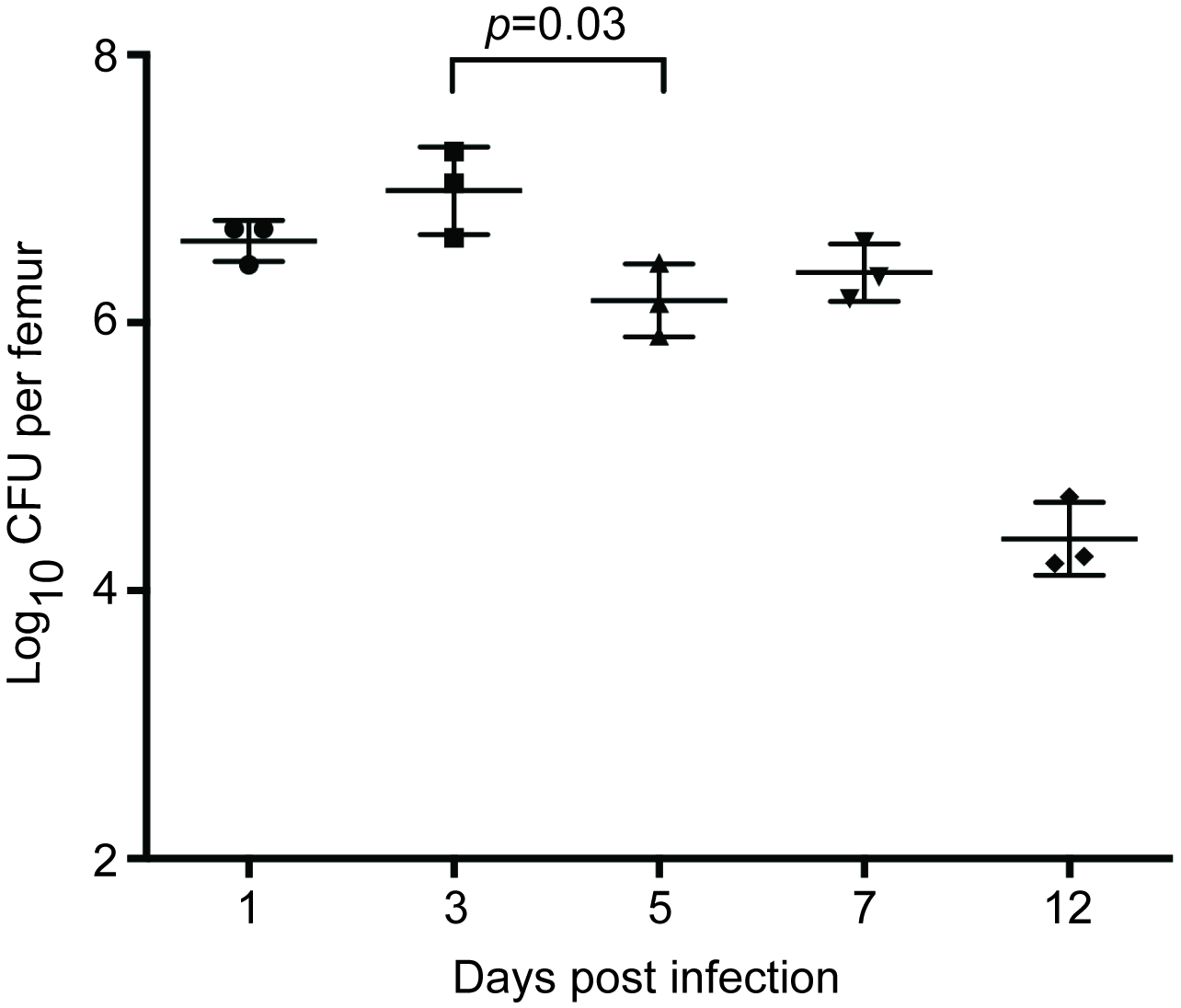

Supplement: S1 Fig — Groups of mice were subjected to osteomyelitis using strain HG003. Infected femurs were harvested at 1, 3, 5, 7, and 12 days post-infection and processed for CFU enumeration (n = 3). Horizontal lines represent the mean. Error bars represent the SD. Significance was determined by Students t test. (TIF) [file ppat.1005341.s005.tif]

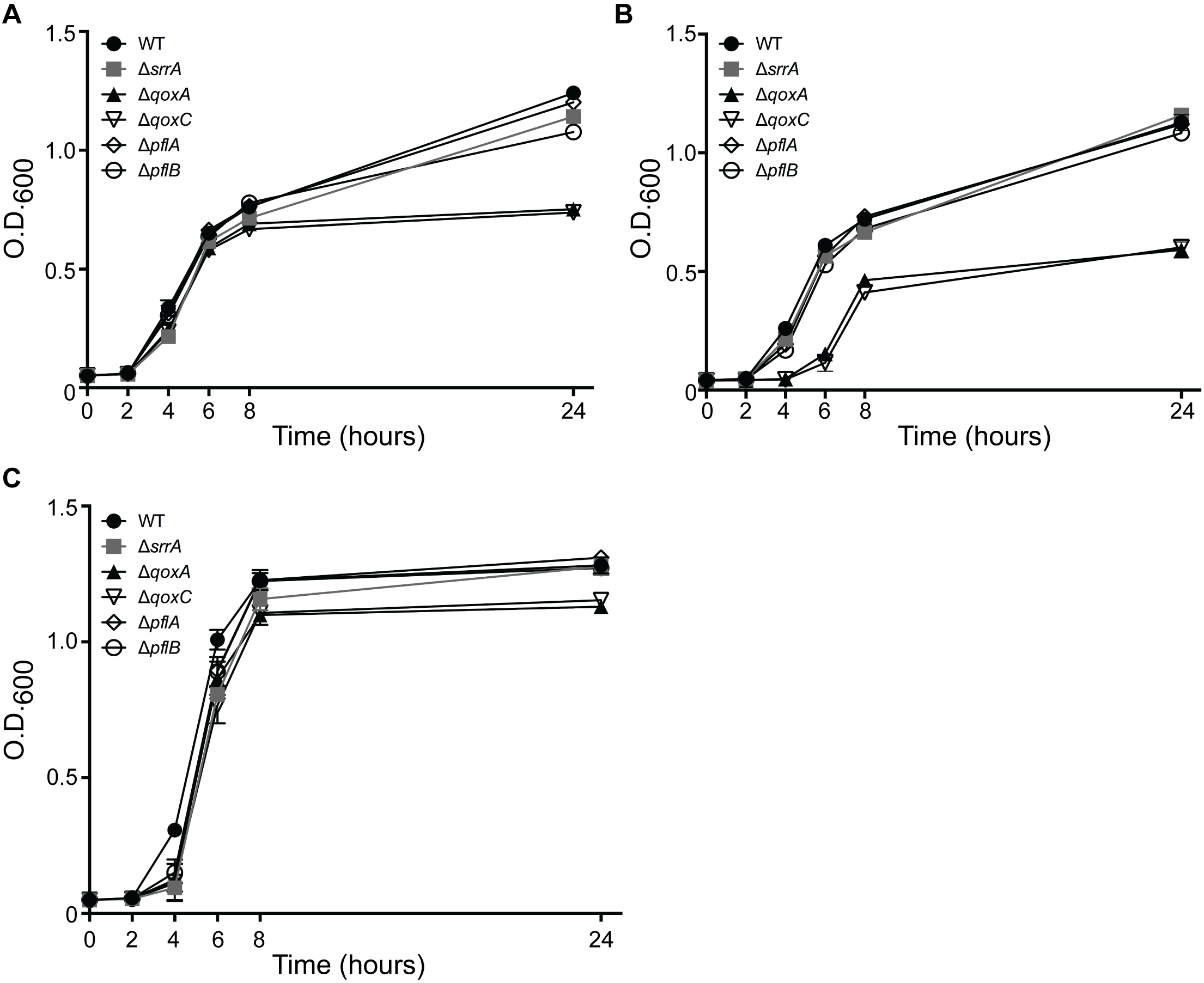

Supplement: S2 Fig — Growth of WT, ΔsrrA, ΔpflA, ΔpflB, ΔqoxA, and ΔqoxC strains was monitored by OD600 with 3 technical replicates at 0, 2, 4, 6, 8, and 24 hours. Data shown is representative of 3 biologically independent experiments. Error bars represent the SEM. (A) Strains grown aerobically in BHI, which served as the in vitro comparator media during TnSeq analysis. (B) Strains grown aerobically in TSB. (C) Strains grown hypoxically in TSB by tightly capping Erlenmeyer flasks. (TIF) [file ppat.1005341.s006.tif]

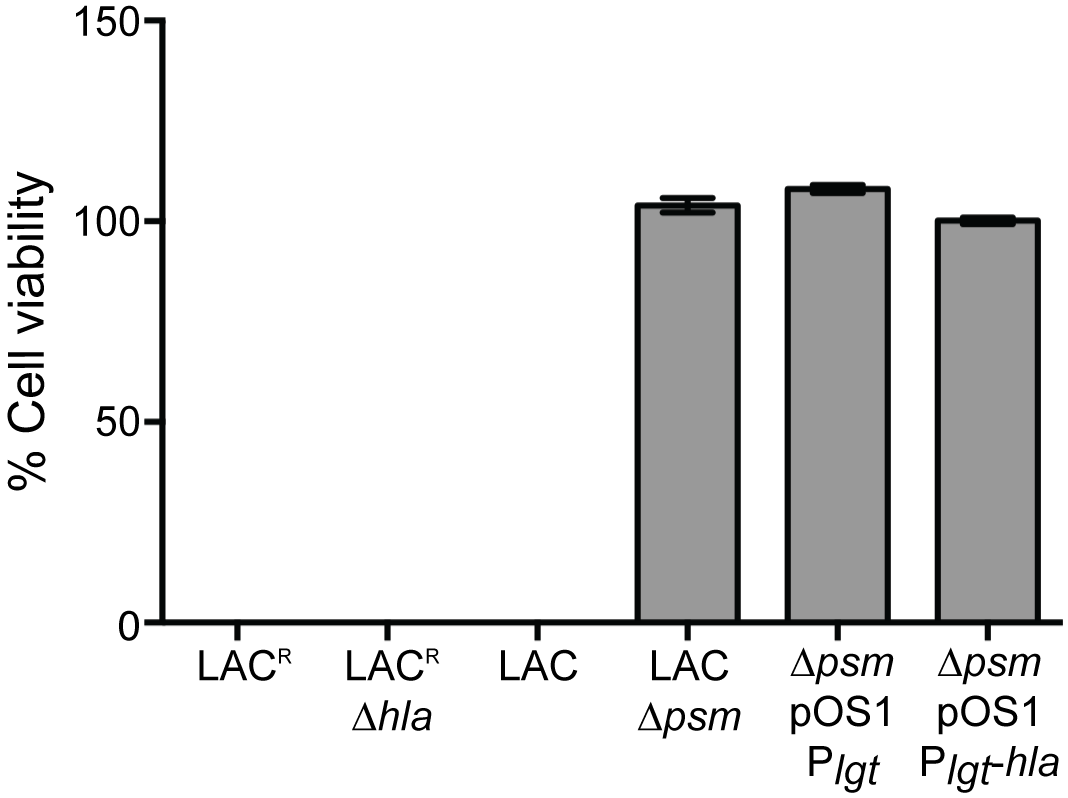

Supplement: S3 Fig — Saos-2 osteoblastic cells were seeded into 96-well plates and cell viability was assessed 24 hours after intoxication with supernatant (30% total media volume) from the indicated strains following hypoxic growth. Results are expressed as percent of RPMI control (n = 10). Error bars represent the SEM. LACR indicates an erythromycin-resistant derivative of LAC used for construction of the hla mutant. (TIF) [file ppat.1005341.s007.tif]

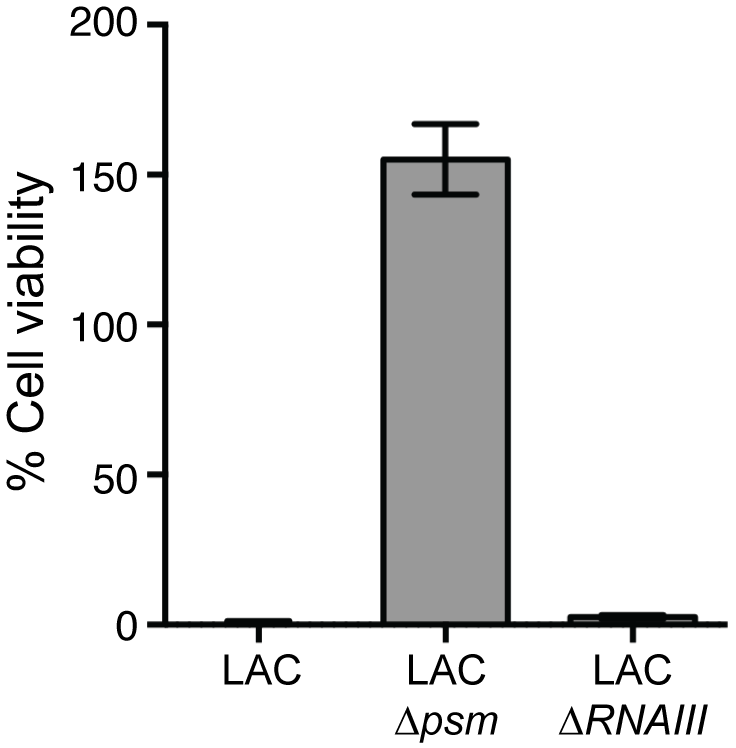

Supplement: S4 Fig — MC3T3 osteoblastic cells were seeded into 96-well plates and cell viability was assessed 24 hours after intoxication with supernatant (30% total media volume) from the indicated strains following hypoxic growth. Results are expressed as percent of RPMI control (n = 10). Error bars represent the SEM. (TIF) [file ppat.1005341.s008.tif]

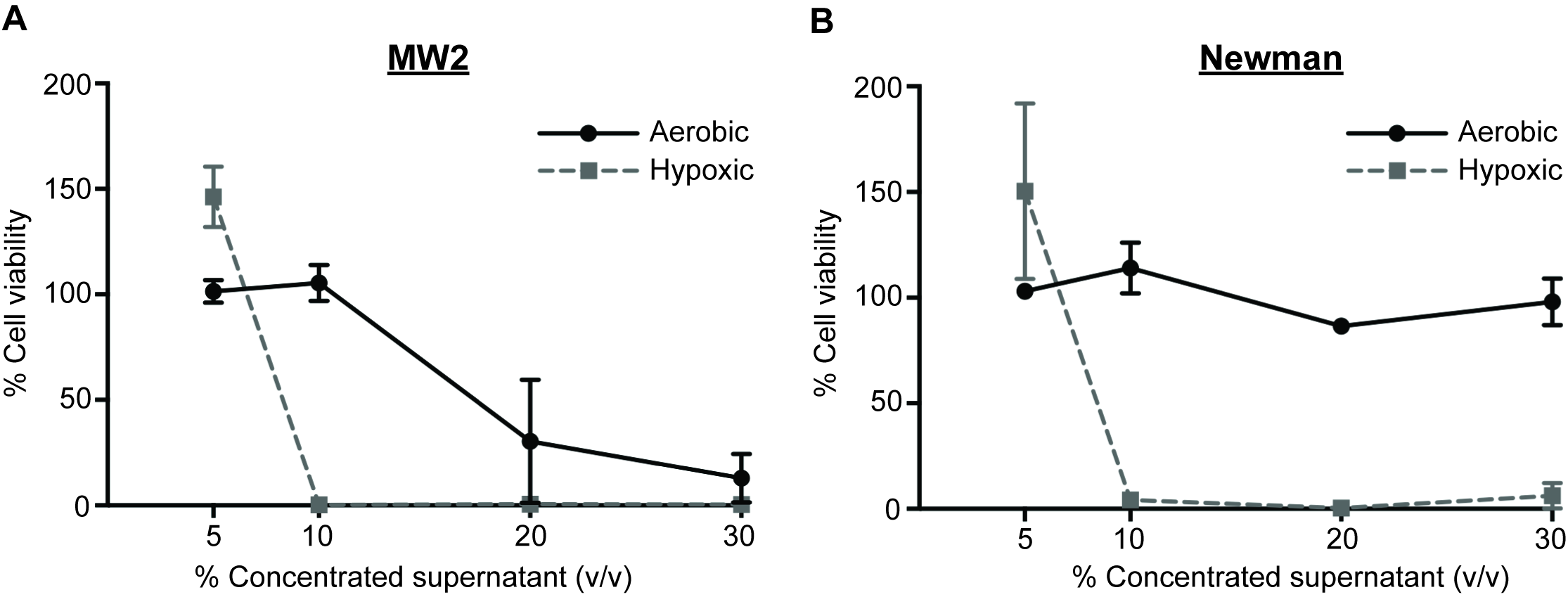

Supplement: S5 Fig — WT supernatants were prepared from strains MW2 (A) and Newman (B) by inoculating 3 colonies into RPMI and 1% casamino acids (CA) and growing for 15 hours either aerobically or hypoxically. MC3T3 murine osteoblastic cells were seeded into 96 well plates 24 hours prior to intoxication with concentrated supernatant or RPMI control. Cell viability was assessed 24 hours later. Results are expressed as percent of RPMI control (n = 10), and are the average of 2 biologic replicates. Error bars represent the SEM. (TIF) [file ppat.1005341.s009.tif]

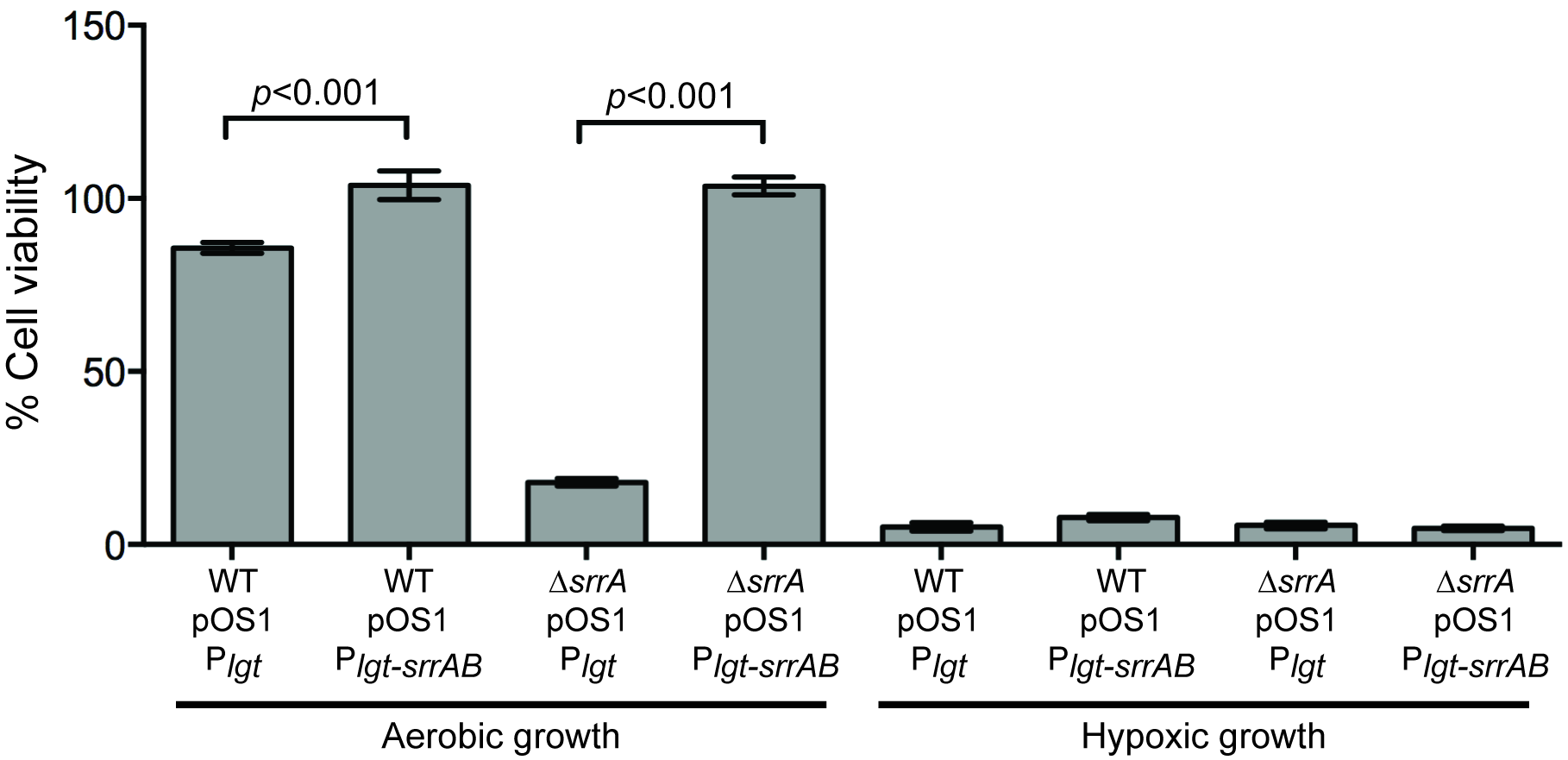

Supplement: S6 Fig — MC3T3 cells were intoxicated with 30% total media volume of RPMI control or concentrated supernatant from the indicated strains after aerobic or hypoxic growth. Cell viability was determined 24 hours after intoxication. Results are expressed as percent of RPMI control (n = 10). Error bars represent the SEM. Significance was determined by Students t test. (TIF) [file ppat.1005341.s010.tif]
